# Computational Studies of Chiral Epoxide Radicals

**DOI:** 10.1021/acs.joc.5c00777

**Published:** 2025-07-16

**Authors:** Kathleen M. Morgan, Lauren A. Brown, Camryn C. Cole, Giavonna K. Cooper, Alajah Nealy, DiJon Seltzer

**Affiliations:** Department of Chemistry, 5785 Xavier University of Louisiana, 1 Drexel Drive, New Orleans, Louisiana 70125, United States

## Abstract

Epoxides are strained heterocycles that are common commodity chemicals and synthetic intermediates. The goal of this study is to understand and compare the reactivity of simple epoxides and their radicals in the gas phase using G4 and W1BD calculations. The epoxides include the parent oxirane and monosubstituted analogs having −CH_3_, –NH_2_, −OH, –F, and –Cl substituents. The C–H bond dissociation energies to form carbon radicals from the various epoxides are reported. Radicals generated on the epoxide ring are nonplanar, and a substituent on the radical carbon has a strong influence on the barrier to invert the radical carbon. Epoxide radicals undergo a competitive ring-opening reaction to form vinoxy radicals, and this process is also influenced by substituents. Calculations on polyfluorinated epoxide radicals were completed, and the barriers to both reactions are elevated in the perfluoro case. These results are largely unchanged when solvent, as incorporated using a polarized continuum model, is included in the calculation. Comparisons to cyclopropyl systems are also made.

## Graphical abstract

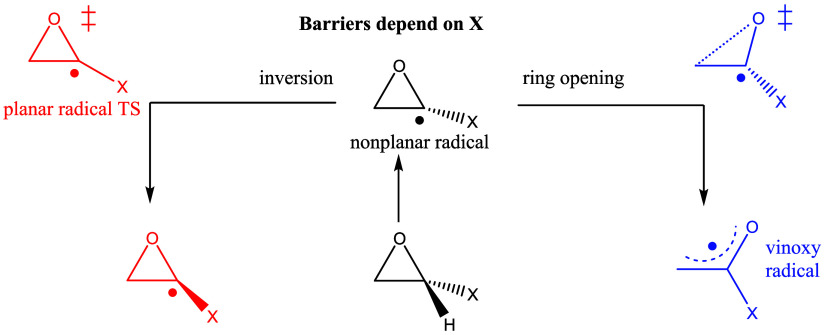

## Introduction

Epoxides are common organic compounds and their reactions are often influenced by strain. Simple epoxides such as ethylene oxide and propene oxide are commodity chemicals. Epoxides are also common synthetic intermediates and are present in numerous drug compounds.^1^ In this study, the influence of substituents on two competing reactions of epoxide radicals, using computational analysis, is described.

Degradation of organic molecules in the gas phase can begin with the formation of a radical.^2^ Radical decomposition of ethylene oxide has been studied previously, and both experiments^3^ and calculations^4^ have been carried out. Barckholtz, Wang, and co-workers published a detailed computational study of the thermal decomposition of ethylene oxide (oxirane) and the oxiranyl radical using G3B3, MP4/6–31+G­(d), and QCISD­(T)/6–31G­(d) methods.^4a^ The mechanism for the unimolecular decomposition of the radical is shown in Figure 1. Cleavage of the CH_2_–O bond, coupled with rotation of the CH_2_ group, leads to the formation of the vinoxy radical. The transition state for this process is the highest energy point on the reaction coordinate, yet this step has the smallest energy of activation.

**Figure 1:**
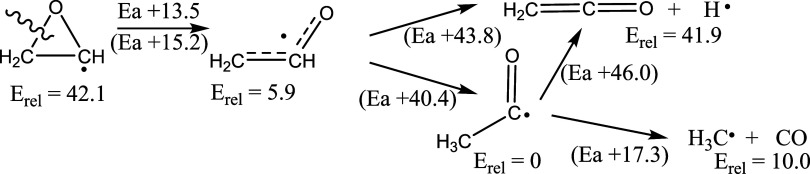
Degradation of the oxiranyl radical, G3B3 calculations, 298 K,^4a^ (CBS-APNO calculations, 298 K),^4b^ kcal/mol.

The authors used RRKM Master Equation Analysis to obtain rate constants, which compared favorably to experiment. More recently, Wang and Bozzelli calculated the radical degradation of ethylene oxide using Δ*H*_f_ obtained from a variety of DFT and compound methods, with similar conclusions.^4b^ One significant difference is that the Δ*H* ^⧧^ for ring opening was found to be higher, +15.2 kcal/mol (CBS-APNO). This paper also provided the barriers for the other reactions, as shown in Figure 1.

FitzPatrick^5^ studied 23 different isomers and four dozen reactions on the C_3_H_5_O potential energy surface, which includes four carbon radicals formed from propene oxide, using coupled cluster calculations and statistical transition state theory modeling. The results were compared to experiment, with good qualitative agreement. There are four C_3_H_5_O epoxide radical isomers, with the relative enthalpies shown in Figure 2.

**Figure 2:**
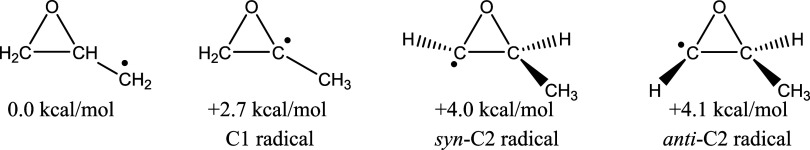
Relative enthalpies of the isomeric epoxide carbon radicals^5^ with nomenclature used in this paper.

The lowest energy isomer has the radical on the carbon outside the ring. There are a variety of interesting reactions that occur from this radical, with the major pathway being facile ring opening to the CH_2_=CHCH_2_O radical, but this chemistry is beyond the scope of the study reported here. When the radical is on the same carbon as the methyl group (C1 radical), the barrier to ring opening to the vinoxy radical is +13.5 kcal/mol. There are two isomers having the radical on C2, the carbon adjacent to the methyl-bearing carbon, as the radical carbon is pyramidal and thus chiral. The barrier to interconvert from *syn* to *anti* is +4.8 kcal/mol, and ring opening to form the *trans* vinoxy radical CH_3_CHCHO has a barrier of +13.4 kcal/mol. FitzPatrick calculated several other possible reaction channels for each of the epoxide radicals, including isomerization of these four radicals and dissociative reactions, and found the barriers to be at least 25 kcal/mol higher than the reactions described above.

The work described in this paper extends these studies to include various substituted epoxides, where the substituents are −CH_3_, –NH_2_, −OH, –F, and –Cl. These substituents were chosen as they are common in organic compounds and are expected to have a range of influence on the inversion and ring-opening processes of interest. In addition, di- and trifluorinated epoxides are considered, and comparisons are made to cyclopropane analogs. The identity, location and number of substituents have a strong influence on both the barrier to racemize the chiral epoxide radical through inversion, and the barrier to open the ring to a vinoxy radical. These reactions are shown in Figure 3 for the C1 radical.

**Figure 3:**
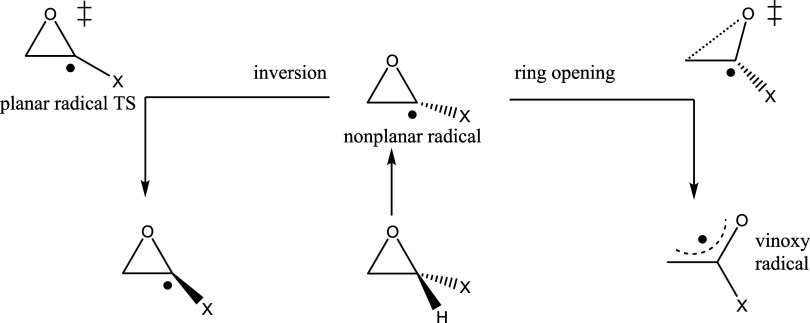
Radical inversion versus ring-opening reactions, C1 radical.

## Results and Discussion

The choice of computational method is especially important for strained compounds, and Kass’s calculations of the cyclopropane bond dissociation energy (BDE)^6^ informed this decision. Kass considered six methods and found reasonably consistent results with CCSD­(T)/aug-cc-pVQZ, W1BD^7^ and G4 ^8^ calculations. In the current work, the structures of all epoxides, cyclopropanes, and radicals were calculated using G4 methodology, which is faster but gives consistently lower BDE. Most calculations were repeated using W1BD methodology, which balances accuracy and efficiency. The W1BD and G4 results presented below are also in good agreement with the computational data noted above.

### Epoxide Structures and Bond Dissociation Energies

The epoxide and three carbon radical isomers were calculated for the parent oxirane and five substituted variations. The three carbon radical isomers are shown in Figure 2 and are the C1, the *syn*-C2, and *anti*-C2 radicals, all having the radical on a ring carbon. In most cases, the epoxides and radicals are rigid and have just one conformation.

For the −NH_2_ and −OH epoxides, three conformations around the C–X bond were explored. In the lowest energy epoxide conformation, the unshared pair of electrons on the heteroatom is oriented *anti* to the C1–O bond in the ring, with a benefit of 2.8–2.9 kcal/mol for the amino group. Two conformations of the alcohol were found, both having the lone pair *anti* to the epoxide C–O bond. The global minimum has the OH hydrogen oriented in toward the epoxide ring, and the conformation having the OH hydrogen oriented out, away from the epoxide ring is +0.2 kcal/mol. The conformation having the OH hydrogen oriented *anti* to the ring oxygen was not found at the G4 or W1BD level of theory.

The lowest energy conformation of the amino and hydroxy C1 radicals has the substituent lone pair aligned so that delocalization with the radical can occur. In the amino series, the global minimum structure has the nitrogen lone pair rotated in toward the ring, with the other two conformations +2.3–2.8 kcal/mol. In the OH series, the conformation having the OH hydrogen out, away from the ring, is now the lowest energy, with the OH *anti* to the ring +0.6 kcal/mol. Both of these conformations have a lone pair rotated in toward the ring as was found in the amine radical. The BDE reported in Table 1 are calculated using the lowest energy conformation of the radical and the epoxide.

**Table 1:** Bond Dissociation Energies, W1BD (G4) Calculations (kcal/mol)

–X	epoxide C1–H	epoxide C2–H (*syn* to –X)	epoxide C2–H (*anti* to –X)	cyclopropane C1–H
–H	104.4 (103.6)			109.0 (108.2)^6^
–CH_3_	102.8 (101.6)	104.1 (103.0)	104.2 (103.1)	106.3 (105.3)
–NH_2_	101.9 (100.8)	102.2 (100.9)	102.7 (101.4)	99.8 (98.8)
–OH	103.3 (102.0)	102.7 (101.5)	103.3 (102.2)	103.1 (102.0)
–F	106.7 (105.5)	105.2 (104.0)	104.9 (103.7)	107.8 (106.7)
–Cl	103.0 (101.8)	105.6 (104.4)	104.8 (103.5)	105.7 (104.5)

The BDE to remove each of the three hydrogens from a monosubstituted epoxide ring, and the C1–H from a cyclopropane ring, to form a carbon radical are shown in Table 1. The epoxide BDE are in most cases lower than those for the cyclopropanes, as the epoxide oxygen stabilizes the adjacent radical. This is reflected in the bond lengths of the O–C1 bond in the epoxide versus its C1 radical, 1.43 to 1.36Å in the parent epoxide (Figure 4). In cyclopropane, the bond also shortens from 1.50Å to 1.46Å, not as pronounced an effect.

**Figure 4:**
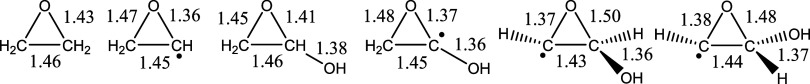
C–O and C–C bond lengths (Å) for oxirane, hydroxyoxirane, and their radicals.

Substituents are known to influence C–H BDE and radical reactivity.^9^ It is expected that alkyl groups and substituents having lone pairs will have an impact on the C1–H BDE (Table 1). Interestingly, the epoxide C2–H BDE are also sensitive to substituents with unshared pairs of electrons. This is in contrast to substituent effects on the cyclopropane C2–H BDE, with BDE values ranging from 109 to 110 kcal/mol (Supporting Information). As an example, the C–O and C–C bond lengths of the parent oxirane, hydroxyoxirane, and their radicals are shown in Figure 4, and the atomic charges and spin, obtained using Natural Population Analysis (NPA),^10^ are shown in Table 2. The epoxide oxygen interacts with the adjacent carbon radical, leading to bond shortening compared to the epoxide. Although the NPA charge on the ring oxygen decreases only slightly between the epoxide and the radicals, this oxygen acquires significant spin in the radicals, with greater spin transfer from carbon to the oxygen(s) in the hydroxyoxiranyl radicals than in the parent. Note that the radical carbon spin is similar in all three hydroxyoxiranyl radicals. In the C1 radical, the two oxygens share the spin while in the C2 radical only the ring oxygen has spin.

**Table 2:** Natural Population Analysis Charges and Spin (W1BD)

	oxirane	oxirane radical	hydroxy oxirane	hydroxy oxirane C1 radical	hydroxy oxirane, *syn* C2 radical	hydroxy oxirane, *anti* C2 radical
Natural Population Analysis Charges						
epoxide O	–0.483	–0.450	–0.502	–0.488	–0.459	–0.466
C1	–0.099	0.095	0.368	0.498	0.308	0.312
C2		–0.154	–0.141	–0.163	0.023	0.036
OH O			–0.705	–0.701	–0.689	–0.689
Natural Population Analysis Spin						
epoxide O		0.141		0.086	0.186	0.182
C1		0.860		0.797	–0.018	–0.030
C2		–0.021		0.018	0.812	0.835
OH O				0.102	0.000	0.008

Formation of the parent epoxide radical is exothermic when H is removed by HO^•^ (−14.5 kcal/mol, G4). Flash photolysis resonance fluorescence experiments on the reaction of ethylene oxide and propene oxide with hydroxyl radical have been reported,^11^ and the reactions are slow, 2−3 orders of magnitude slower than reaction with aldehydes.^2b^ A preparative route to epoxide radicals, especially with any regioselectivity, would require other radical generation methods.

### Epoxide Radical and Cyclopropyl Radical Inversion Versus Ring-Opening Reactions

The carbon radicals formed on epoxide and cyclopropane rings are not planar. The sum of the bond angles around the C1 radical carbon is shown in Table 3. A planar radical angle sum is 360°, and the more the angle sum deviates from 360° the more pyramidal the radical. A general trend seen in Table 3 is that the more pyramidal radicals have higher barriers to invert the radical. The C1 epoxide radicals with −OH and –F substituents have very high inversion barriers, 19.8 and 25.4 kcal/mol, suggesting that these chiral radicals will racemize slowly. In contrast, the cyclopropyl C1 radicals all have fairly low barriers to invert the radical.

**Table 3:** Pyramidalization of C1 Radicals and Δ*H* ^⧧^ to Invert Versus Ring Open, W1BD (G4) Calculations (kcal/mol)

	angle sum, epoxide C1 radical	Δ*H* ^⧧^ to invert epoxide C1 radical	Δ*H* ^⧧^ to open epoxide C1 radical to vinoxy		angle sum, cyclopropyl C1 radical	Δ*H* ^⧧^ to invert cyclopropyl C1 radical
			TS-A	TS-B		
–H	317.7 (316.6)	5.2 (5.3)	14.4 (14.0)	15.5 (16.1)	328.0 (327.9)	1.4 (1.5)
–CH_3_	320.4 (320.1)	7.3 (7.2)	14.0 (13.7)	15.9 (16.4)	329.9 (331.4)	2.8 (2.8)
–NH_2_	308.7 (307.3)	13.7 (13.6)	13.0 (12.8)	16.8 (17.1)	314.0 (313.2)	8.7 (8.8)
–OH	310.7 (309.7)	19.8 (19.7)	12.3 (12.3)	17.0 (17.4)	313.9 (315.8)	9.7 (9.8)
–F	309.5 (309.6)	25.4 (25.4)	12.5 (12.8)	17.1 (17.7)	311.2 (312.6)	11.2 (11.3)
–Cl	315.9 (314.5)	13.6 (13.3)	13.7 (14.1)	16.7 (17.6)	322.6 (322.0)	(5.0)

There are two diastereomeric ring-opening transition states for the parent and C1-substituted oxiranyl radicals, as shown in Figure 5. Both transition states have a roughly planar CH_2_ carbon and pyramidal radical CHO carbon. They differ in the direction of the twist of the CH_2_ with respect to the pyramidalized radical. In the lower energy structure, TS-A, the CH_2_ hydrogen *syn* to the radical rotates to become *trans* to the oxygen in the vinoxy product. The second structure, TS-B, is higher energy for the parent and C1-substituted examples, and here the CH_2_ hydrogen *syn* to the radical rotates to become *cis* to the oxygen in the vinoxy product. Substituent effects on the C1 ring opening processes are small when compared with their influence on radical inversion. With regard to the cyclopropyl radical ring opening, the barrier to open the ring to an allyl radical is high, over 20 kcal/mol in all cases (Supporting Information).

**Figure 5:**
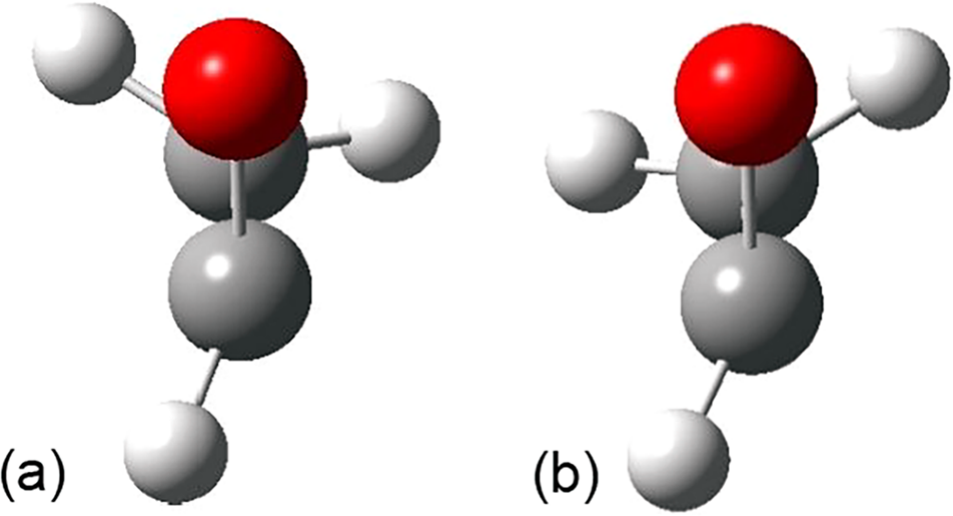
Diastereomeric ring opening transition states, oxiranyl radical (a) TS-A; (b) TS-B.

Table 4 includes radical inversion versus ring opening barriers for the *syn*- and *anti-*C2 epoxide radicals. In the substituted C2 epoxide radicals, there are four diastereomeric ring-opening transition states, TS-A and TS-B for both the *syn-*C2 and *anti*-C2 radicals. Shown in Figure 6 are two of these, both TS-A, that connect the *syn*-C2 epoxide radical to the *trans* vinoxy radical (Figure 6a), and the *anti*-C2 epoxide radical to the *cis* vinoxy radical (Figure 6b), as confirmed using Intrinsic Reaction Coordinate (IRC) calculations. The ring opening barriers in Table 4 are the difference in enthalpy of the transition state and the epoxide radical diastereomer that connects to that transition state.

**Figure 6:**
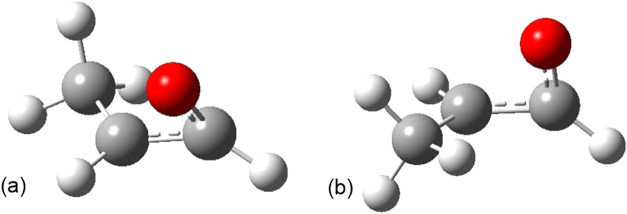
Diastereomeric epoxide radical ring-opening transition states, TS-A. (a) C2 radical *syn* to substituent going to *trans* vinoxy and (b) C2 radical *anti* to substituent going to *cis* vinoxy.

**Table 4:** ΔH^⧧^ for C2 Radicals to Invert Versus Ring Open, W1BD (G4) Calculations (kcal/mol)

	Δ*H* ^⧧^ to invert epoxide C2 radical, from *syn*	Δ*H* ^⧧^ to invert epoxide C2 radical, from *anti*	Δ*H* ^⧧^ to open epoxide C2 *syn* radical to vinoxy		Δ*H* ^⧧^ to open epoxide C2 *anti* radical to vinoxy	
			TS-A, *trans*	TS-B, *cis*	TS-A, *cis*	TS-B, *trans*
–CH_3_	5.0 (5.0)	4.9 (4.9)	13.4 (13.0)	16.2 (16.7)	16.4 (15.9)	13.8 (14.0)
–NH_2_	(3.9)	(3.4)	2.6 (2.6)	6.3 (5.5)	(8.6)	2.3 (2.0)
–OH	4.0 (4.0)	3.3 (3.3)	7.6 (4.2)	12.7 (11.1)	17.1 (18.5)	7.2 (6.8)
–F	3.9 (3.9)	4.2 (4.2)	13.2 (13.3)	20.0 (21.1)	20.2 (20.1)	13.5 (13.8)
–Cl	4.1 (4.2)	4.9 (5.0)	11.1 (10.7)		17.9 (17.2)	11.7 (11.7)

The barrier to inversion for the C2 epoxide radicals is not sensitive to the substituent, and similar to the parent oxiranyl radical. The transition state to invert the C2 aminooxirane radicals was difficult to calculate; while the structure was successfully obtained using G4 calculations, only a second-order saddle point could be found at the W1BD level. The two imaginary vibrational modes correspond to inversion of the C2 radical and ring opening to the vinoxy radical. This is not altogether surprising, as the C1–O bond in the C2 amino radicals is especially long, 1.57Å (*syn*) and 1.55 Å (*anti*), which is consistent with the low barrier for ring opening.

The C2 substituent affects the ring-opening reactions significantly. The reactions that form *trans* vinoxy products have lower barriers than found for the parent ring opening. However, several of the barriers that lead to *cis* products are much higher, especially in the case of fluorine. The transition states leading to the *trans* products would be expected to reflect the lower energy of the *trans versus cis* vinoxy products, but the difference in energy between the *cis* and *trans* vinoxy products is small, for example less than 1 kcal/mol with the methyl or fluoro substituents, and does not fully account for this finding. Two ring-opening transition states could not be found, one with the amine at the W1BD level and one with chlorine. In both cases, attempts at optimization either led to an inverted radical carbon, or a second-order saddle point.

Combining the results of Tables 3 and 4 leads to an interesting possibility. A fluorine substituent on the same carbon as the epoxide radical raises the barrier to racemize via inversion, and fluorines on the adjacent carbon raise the barrier to ring open to the *cis* vinoxy but not the *trans* vinoxy. What happens in cases having fluorine on both C1 and C2? The reactions of three difluorooxirane radical isomers and the trifluorooxirane radical were considered, with the results in Table 5. TS-A for the 1,1-difluorooxirane radical could not be obtained as attempts at optimization led either to inversion of the radical carbon or a second-order saddle point. The calculations suggest that the epoxide radical having trifluoro substitution is likely to be stabilized to both racemization and ring-opening reactions.

**Table 5:** Polyfluorooxirane BDE, and Δ*H* ^⧧^ for Inversion *Versus* Ring Opening, W1BD (G4) Calculations (kcal/mol)

	1,1-difluorooxirane	*Cis*-1,2-difluorooxirane	*Trans*-1,2-difluorooxirane	trifluorooxirane
C–H BDE	106.2 (104.7)	107.0 (105.4)	107.9 (106.3)	108.1 (106.2)
angle sum, epoxide radical	317.0 (316.1)	306.6 (306.4)	309.3 (309.7)	306.6 (306.5)
Δ*H* ^⧧^ to invert epoxide radical	3.8 (3.8)	21.0 (20.9)	21.8 (21.7)	20.3 (20.3)
Δ*H* ^⧧^ to open epoxide radical to vinoxy, TS-A		18.2 (19.3)	13.5 (13.9)	18.7 (19.3)
Δ*H* ^⧧^ to open epoxide radical to vinoxy, TS-B	18.4 (18.7)	14.7 (15.1)	19.8 (20.5)	16.9 (17.0)

Persistent radicals, defined by Ingold as those with lifetimes of seconds to hours,^12^ have been known since Gomberg’s report of triphenylmethyl radical in 1900.^13^ Stable radicals are even less reactive; they can be isolated in pure form and handled using standard techniques.^12^ Persistent and stable radicals have received much interest in recent years, and have many potential applications in drug development and materials, among others.^14^ The substituted epoxide radicals are not likely to fall into these categories, but they may still be valuable.

The information learned in the current study on the use of substituents to decrease the rates of both racemization and ring-opening reactions of oxiranyl radicals may lead to development of a known reaction. Ziegler generated alkyl oxiranyl radicals via the photochemical degradation of thiohydroxamate and related esters at room temperature or 0 °C in THF.^15^ Although the radicals racemized rapidly, they cyclized into a tethered indole or alkene with good stereoselectivity, perhaps due to stereoelectronic effects and/or strain. In some cases, ring opening products formed competitively. Intermolecular reactions of alkyl oxiranyl radicals with alkenes were also attempted, with a 9:1 ratio of epoxide diastereomers, likely due to different reaction rates for the *syn* versus *anti* radicals. This chemistry has not yet been applied widely despite its promise.

### Solvent Effects

Solvent is known to influence radical reactions.^16^ To evaluate the effects of solvent on the barriers to invert versus ring open the epoxide radicals, calculations were carried out with solvent incorporated using the Polarized Continuum Model^17^ and applied to the parent oxirane and trifluorooxirane series of reactions. In this model, the solute is held in a cavity surrounded by solvent that is treated as a continuum, without discrete solvent–solute interactions. As shown in Table 6, solvent has a minimal impact on the results for the parent epoxide racemization and ring opening via TS-A. This is not surprising as the gas-phase dipole moments of the radical, radical inversion transition state, and TS-A ring-opening transition state are similar, 1.74D, 1.66D and 1.95D, respectively. The gas-phase dipole moment for TS-B is 2.27D and the barrier for this process decreases as solvent dielectric constant increases. In the trifluorooxirane series, the barrier to invert the radical is constant, but both barriers to ring opening decrease by a few kcal/mol in the more polar solvents. Here, the gas-phase dipole moments for the radical and radical inversion transition state are similar, 0.64D and 0.95D, while the dipole moments for the ring-opening transition states are quite different, 2.21D for TS-A and 2.56D for TS-B, and will be stabilized in the more polar solvents. Still, the calculations suggest that chiral epoxide radicals could exist in less polar solvents as well as the gas phase.

**Table 6:** PCM Solvent Effects on Epoxide Radical Inversion *Versus* Ring-Opening Reactions, G4 Calculations (kcal/mol)

	ethylene oxide series				trifluoroethylene oxide series			
	C–H BDE	Δ*H* ^⧧^ to invert	Δ*H* ^⧧^ to ring open		C–H BDE	Δ*H* ^⧧^ to invert	Δ*H* ^⧧^ to ring open	
			TS-A	TS-B			TS-A	TS-B
gas phase ε = 1.0	103.6	5.3	14.0	16.1	106.2	20.3	19.3	17.0
cyclohexane ε = 2.02	103.7	5.2	14.0	15.6	107.0	20.1	18.6	15.9
benzene ε = 2.27	103.8	5.2	14.0	15.5	107.0	20.1	18.5	15.7
chlorobenzene ε = 5.70	104.0	5.2	14.1	15.0	107.6	20.0	17.2	14.8
THF ε = 7.43	104.0	5.2	14.1	14.9	107.7	20.0	17.1	14.6
methanol ε = 32.61	104.1	5.2	14.1	14.6	108.0	19.9	16.9	14.1
acetonitrile ε = 35.69	104.1	5.2	14.1	14.5	108.0	19.9	16.9	14.1
water ε = 78.36	104.1	5.2	14.2	14.5	108.1	19.9	16.8	14.0

## Conclusions

The formation and reactions of chiral carbon radicals formed on epoxide rings were studied using W1BD and G4 computational methods. Substituents can have a significant impact on two competing reactions for these radicals. If the substituent has a lone pair of electrons and is on the same carbon as the radical, the barrier to invert the chirality of the pyramidal radical increases, with oxygen and fluorine substituents having the greatest impact. Substituents on the carbon adjacent to the radical carbon can increase the barrier to open the epoxide ring to a *cis*-vinoxy radical, with fluorine again having the strongest effect. However, ring opening to the *trans*-vinoxy radical is faster than the parent. The carbon radical formed from 1,1,2-trifluorooxirane is found to have high barriers for both radical inversion and ring opening, all at least 16.9 kcal/mol. These gas-phase results are largely unaffected when solvent is included in the calculations. The calculations suggest that a new family of chiral epoxide radicals with extended lifetimes may exist.

### Computational Methods

Calculations were carried out either on a PC running Gaussian 16W rev B.01 ^18^ interfaced with GaussView 6.1.1, or on a Linux system running Gaussian 16 rev A.03 ^19^ interfaced with GaussView 6.0.16. In some cases, conformational analysis was performed using Spartan’20, Version 1.1.4 ^20^ on a PC. All minima obtained in Gaussian, at 298 K, were verified to have zero imaginary vibrational frequencies, and transition states all had one. IRC calculations were performed on some ring-opening transition states, especially to confirm which epoxide radical isomer connects to which vinoxy radical, and in all cases, the vibrational mode was animated in GaussView. In the PCM solvent calculations, structures were reoptimized in the solvent continuum.

## Supplementary Material



## Data Availability

The data underlying this study are available in the published article and its Supporting Information.

## Notes

Additional data on substituted cyclopropyl radicals as well as Cartesian coordinates and calculated enthalpies for all compounds (PDF)
