# Supplementary material for: Computational Studies of Chiral Epoxide Radicals
Source: J Org Chem. 2025 Jul 16;90(29):10235–41. doi: 10.1021/acs.joc.5c00777 (PMC12305647; doi:10.1021/acs.joc.5c00777)
Supplement: Supplementary file 1 [file jo5c00777_si_001.pdf]

Supporting Information

# Computational Studies of Chiral Epoxide Radicals

Kathleen M. Morgan,* Lauren A. Brown, Camryn C. Cole, Giavonna K. Cooper, Alajah Nealy, DiJon Seltzer

Department of Chemistry, Xavier University of Louisiana, 1 Drexel Drive, New Orleans LA 70125 USA, kmmorgan@xula.edu

Table of Contents

Table S-1. BDE of cyclopropyl C2 radicals; ΔH‡ for radical inversion and ring opening . S-2

Calculated enthalpies (Hartrees) and cartesian coordinates from W1BD optimizations
Epoxides . . . . . . . . . . . . . . . . . . . . . . . . . . . . . . . . . S-3

Cyclopropanes . . . . . . . . . . . . . . . . . . . . . . . . . . . . . S-6

C1 epoxide radicals . . . . . . . . . . . . . . . . . . . . . . . . . . . S-8

C1 cyclopropyl radicals . . . . . . . . . . . . . . . . . . . . . . . . S-10

C1 epoxide radical inversion transition states . . . . . . . . . . . . . . . . . S-12

C1 cyclopropyl radical inversion transition states . . . . . . . . . . . . . . . S-14

C1 epoxide radical ring opening transition states . . . . . . . . . . . . . . . S-15

C1 cyclopropyl radical ring opening transition states . . . . . . . . . . . . . S-20

C2 epoxide radicals . . . . . . . . . . . . . . . . . . . . . . . . . . . S-20

C2 cyclopropyl radicals . . . . . . . . . . . . . . . . . . . . . . . . .S-23

C2 epoxide radical inversion transition states . . . . . . . . . . . . . . . . . S-25

C2 cyclopropyl radical inversion transition states . . . . . . . . . . . . . . .S-26

C2 epoxide radical ring opening transition states . . . . . . . . . . . . . . .S-27

C2 cyclopropyl radical ring opening transition states . . . . . . . . . . . . . S-33

Table S-1. BDE of cyclopropyl C2 radicals; ΔH$^{‡}$ for radical inversion and ring opening, W1BD (G4) calculations (kcal/mol)

| | Cyclopropane | Methyl cyclopropane | Fluoro cyclopropane | 1,1,2-trifluoro cyclopropane |
|---|---|---|---|---|
| BDE, *syn*-C2H | 109.0 (108.2) | 109.2 (108.1) | 110.1 (108.9) | 108.2 (106.4) |
| BDE, *anti*-C2H | | 109.0 (107.9) | 108.9 (107.8) | |
| ΔH$^{‡}$ to invert C2 *syn* radical | 1.4 (1.5) | 1.3 (1.4) | 0.0 (0.1) | 6.5 (6.5) |
| ΔH$^{‡}$ to invert C2 *anti* radical | | 1.5 (1.6) | 1.1 (1.2) | |
| ΔH$^{‡}$ to open C2 *syn* radical | 22.6 (22.8) | 21.3 (21.7)<br>23.9 (24.2) | 19.9 (20.3)<br>22.3 (22.7) | 22.7 (23.8)<br>19.6 (19.9) |
| ΔH$^{‡}$ to open C2 *anti* radical | | 23.1 (23.5)<br>20.5 (20.6) | 22.9 (23.5)<br>16.8 (17.0) | |

**Epoxides**
Ethylene oxide
H G4 -153.69620
H W1BD -153.794753
O,0,0.,0.,1.176964365
C,0,0.7316017341,0.,-0.0490431838
C,0,-0.7316017341,0.,-0.0490431838
H,0,1.2665732703,-0.917530267,-0.2697194986
H,0,1.2665732703,0.917530267,-0.2697194986
H,0,-1.2665732703,-0.917530267,-0.2697194986
H,0,-1.2665732703,0.917530267,-0.2697194986

Methyloxirane
H G4 -192.98068
H W1BD -193.09598
C,0,-1.0403144314,0.6154821389,-0.0568283115
C,0,0.151898137,-0.039464442,0.4847936042
O,0,-0.8269260313,-0.7868076543,-0.2470827658
H,0,-1.8646116693,0.874482505,0.5997077383
H,0,-0.9510435364,1.2219522504,-0.9527865135
H,0,0.1537676368,-0.2619613408,1.5492300626
C,0,1.5068276509,0.1017291277,-0.1479365045
H,0,2.0719091926,0.9039564866,0.3310871554
H,0,2.0790107068,-0.8214269621,-0.0422809475
H,0,1.4141923443,0.3265488907,-1.2102155177

Aminooxirane, global minimum, $NH_2$ lone pair *anti* to ring
H G4 -209.03125
H W1BD -209.16663
C,0,1.0121043144,-0.6023041374,0.0033161522
C,0,-0.1940809855,0.0704176639,0.4637022393
O,0,0.8008000268,0.7853589964,-0.3067867372
H,0,1.8245953599,-0.8129207086,0.6916195147
H,0,0.9447913397,-1.2814495902,-0.842418082
H,0,-0.2475051083,0.3974190929,1.4962758377
N,0,-1.4648697187,-0.2059330651,-0.0885961149
H,0,-2.079295295,0.5962565566,-0.0284847297
H,0,-1.4000379333,-0.4867558085,-1.0587090801

Aminooxirane, +2.7 kcal/mol, $NH_2$ lone pair rotated “out” from ring
H G4 -209.02675
H W1BD -209.16225
C,0,-1.0263690769,0.6197684147,-0.0487577988
C,0,0.1882096173,-0.034444879,0.457926975
O,0,-0.7964925121,-0.7950313861,-0.2161569366
H,0,-1.8450342605,0.8806511318,0.6154204149
H,0,-0.9658468069,1.2031833425,-0.962834661
H,0,0.2639440923,-0.2292825581,1.5244820096
N,0,1.4683724527,0.0120553971,-0.1638878445
H,0,1.4176514794,0.3595434608,-1.113058838
H,0,2.1162610146,0.5818760763,0.3639836793

Aminooxirane, +2.9 kcal/mol, $NH_2$ lone pair rotated “in” towards ring
H G4 -209.02668
H W1BD -209.16208
C,0,-0.9863070042,0.6337990609,-0.0189205728
C,0,0.1874200108,-0.0996684972,0.4500754191
O,0,-0.8470255969,-0.7628256927,-0.2836217612
H,0,-1.7734231607,0.9079442239,0.6761930378
H,0,-0.8771068031,1.2765880049,-0.8866341479
H,0,0.2132688635,-0.4179354344,1.4905572512
N,0,1.4291876347,0.0785728857,-0.2214332527
H,0,1.7917135014,1.0150527612,-0.1073223153
H,0,2.1299935545,-0.5816863122,0.0857303419

Hydroxyoxirane, OH rotated “in” towards ring
H G4 -228.90527
H W1BD -229.07039
C,0,1.4429153799,-1.4576679212,-0.8638920191
C,0,1.856019249,-0.3742769367,0.0261904467
O,0,0.5116230338,-0.8034399494,0.0359478952
H,0,1.2680771994,-1.2798176468,-1.9198287659
H,0,1.6915246864,-2.4806331234,-0.5984734873
H,0,2.030896422,0.6235815601,-0.3594706753
O,0,2.6020536405,-0.560428155,1.1702262056
H,0,2.3553940989,-1.4035338576,1.5677258801

Hydroxyoxirane, +0.2 kcal/mol, OH rotated “out” from ring
H G4 -228.90514
H W1BD -229.07007
C,0,2.2161527871,-1.0358050054,-1.2517782928
C,0,1.842562467,-0.4348213291,0.0177072277
O,0,1.0549682535,-1.4826148303,-0.5200405071
H,0,2.0108108318,-0.5261883102,-2.1867898569
H,0,3.0459437601,-1.7333219727,-1.2564150172
H,0,1.3367266781,0.5269625442,0.0526440749
O,0,2.6239595214,-0.7215302134,1.119249938
H,0,2.0846651311,-0.6490744831,1.9139769233

Fluorooxirane
H G4 -252.92837
H W1BD -253.13579
C,0,0.9768300349,-0.6162686241,-0.0231538719
C,0,-0.2184453306,0.0626991625,0.4494701111
O,0,0.7205479523,0.7982384178,-0.2482688844
H,0,1.7960370827,-0.8352171181,0.6516893656
H,0,0.8958685499,-1.2325204461,-0.9102079708
H,0,-0.401213384,0.3357166485,1.482715865
F,0,-1.3963509052,-0.1522500406,-0.1991086146

1,1-Difluorooxirane

H G4 -352.16339
H W1BD -352.47955
C,0,0.0441021454,1.0837102563,0.
C,0,1.4877051232,1.0143114016,0.
O,0,0.693512005,2.2709795376,0.
H,0,2.0142442496,0.8251687011,-0.9264485136
H,0,2.0142442496,0.8251687011,0.9264485136
F,0,-0.6860753564,0.7918521512,-1.0863364462
F,0,-0.6860753564,0.7918521512,1.0863364462

*Cis*-1,2-difluorooxirane
H G4 -352.15508
H W1BD -352.47128
C,0,-1.1509100377,-0.3319301576,-0.024216801
C,0,0.3040369871,-0.3360987128,-0.0240308842
O,0,-0.4204541884,0.8697924195,-0.0403267983
H,0,-1.7798378146,-0.5370113974,-0.8826436066
H,0,0.9320657139,-0.5439620316,-0.8823951632
F,0,0.9442514595,-0.6168468593,1.1318460515
F,0,-1.7927918396,-0.6098155208,1.1315086218

*Trans*-1,2-difluorooxirane
H G4 -352.15795
H W1BD -352.47410
C,0,-0.3643488594,-3.2015399997,-0.0138446561
C,0,1.0854068188,-3.2057015329,0.0137651112
O,0,0.3635538314,-1.9987821189,0.0004693475
H,0,-0.9426583381,-3.4124464344,-0.905309372
H,0,1.6625524486,-3.4198371399,0.9051740191
F,0,1.7830899386,-3.4748687166,-1.1150460123
F,0,-1.0633784299,-3.4682740075,1.1147915625

Trifluorooxirane
H G4 -451.38874
H W1BD -451.81361
C,0,-0.0182901608,1.7036527324,0.0140354204
C,0,1.4297395364,1.7335636902,0.0020184651
O,0,0.7447745207,2.9232685648,0.0179689358
H,0,-0.615150846,1.5171078352,0.8983350493
F,0,-0.6897890126,1.4523698033,-1.1206871982
F,0,2.1320599472,1.4556515327,-1.0948939211
F,0,2.1708254452,1.4598584013,1.0797408287

Chlorooxirane
H G4 -613.18609
H W1BD -614.70598
C,0,0.7751896969,-0.2310956261,-0.0183075295
C,0,2.231579083,-0.2560361142,-0.0077152312
O,0,1.5783294571,0.9713432172,-0.0213553985
H,0,0.2347560087,-0.4263476957,-0.9376071641
H,0,0.2482748126,-0.423636507,0.9079194279

Cl,0,3.1085663559,-0.6949040566,1.4758915306
H,0,2.8233210159,-0.5074709976,-0.8776518052

**Cyclopropanes**

Cyclopropane
H G4 -117.78584
H W1BD -117.83571
C,0,0.7519876437,-0.4341581414,0.
C,0,-0.7519786577,-0.4341542333,0.
C,0,-0.0000011943,0.8683117492,0.
H,0,1.2603694493,-0.7276719023,0.9074152808
H,0,1.2603694493,-0.7276719023,-0.9074152808
H,0,-1.2603642375,-0.727667446,0.9074118912
H,0,-1.2603642375,-0.727667446,-0.9074118912
H,0,-0.0000051082,1.4553431616,-0.9074119063
H,0,-0.0000051082,1.4553431616,0.9074119063

Methylcyclopropane
H G4 -157.06579
H W1BD -157.13226
C,0,0.3894133832,-2.0040768503,0.0072500668
C,0,1.8972722744,-2.0038709513,0.0072474587
C,0,1.1431659994,-0.7028489185,-0.0183729968
H,0,-0.1140693137,-2.3103454321,-0.9001906717
H,0,-0.1219161404,-2.2897842951,0.9159423814
H,0,2.4008355883,-2.3100024943,-0.9001942574
H,0,2.4086820064,-2.2894389091,0.9159379018
H,0,1.1430910951,-0.148295159,0.912669767
C,0,1.1430421736,0.157676181,-1.2581455564
H,0,0.2605679145,0.7998561108,-1.2964507833
H,0,2.0253398969,0.8000982998,-1.2964563813
H,0,1.1431237623,-0.4577301218,-2.1599139786

Hydroxycyclopropane, OH rotated “out” from ring
H G4 -192.98307
H W1BD -193.09927
H,0,0.7119343747,-1.236027385,-1.0729317755
C,0,0.910293598,-0.7416158163,-0.1329919407
C,0,0.8895599841,0.7726424645,-0.136030825
C,0,-0.2349449696,-0.0116024807,0.4778613607
H,0,1.6052170012,-1.2536221438,0.5169665104
H,0,0.6863520404,1.2558838645,-1.0812779927
H,0,1.5688067383,1.3053936899,0.5149152698
H,0,-0.3155195745,-0.0156732887,1.5604955901
O,0,-1.4593928806,-0.1086611276,-0.2010046799
H,0,-1.928976312,0.7252102231,-0.0992485172

Hydroxycyclopropane, +1.9 kcal/mol, OH rotated “in” towards ring
H G4 -192.97995
H W1BD -193.09627
H,0,-0.6140086914,-1.1402687272,1.2535082514

C,0,0.3067729561,-0.8560439976,0.7606043982
C,0,0.3067729561,-0.8560439976,-0.7606043982
C,0,0.3097826009,0.432719457,0.
H,0,1.2018869373,-1.1614009066,1.2845637121
H,0,-0.6140086914,-1.1402687272,-1.2535082514
H,0,1.2018869373,-1.1614009066,-1.2845637121
H,0,1.2201451544,1.0150895758,0.
O,0,-0.7900233992,1.3032627754,0.
H,0,-1.6015647606,0.7868724545,0.

Aminocyclopropane, NH lone pair rotated “in” towards ring
H G4 -173.11068
H W1BD -173.19717
H,0,0.6021170634,-1.1505830455,-1.2471420773
C,0,-0.3205598319,-0.8816980481,-0.7536112497
C,0,-0.3205598319,-0.8816980481,0.7536112497
C,0,-0.3225640265,0.417269093,0.
H,0,-1.222344811,-1.1781797022,-1.2702287388
H,0,0.6021170634,-1.1505830455,1.2471420773
H,0,-1.222344811,-1.1781797022,1.2702287388
H,0,-1.2640002694,0.9627357277,0.
N,0,0.903017552,1.1771656137,0.
H,0,0.9582189507,1.7749115772,-0.8161142407
H,0,0.9582189507,1.7749115772,0.8161142407

Aminocyclopropane, +1.9 kcal/mol NH lone pair rotated “out” from ring
H G4 -173.10740
H W1BD -173.19409
H,0,-0.7285273594,1.2110644087,-1.1410252485
C,0,-0.9266407591,0.7614145958,-0.1772008498
C,0,-0.9183326278,-0.7500577245,-0.1009137207
C,0,0.21218378,0.0264607999,0.4912782663
H,0,-1.6335822926,1.3035552505,0.4362900116
H,0,-0.722587423,-1.2952803569,-1.0152967103
H,0,-1.6173328474,-1.2410240081,0.5610448657
H,0,0.2374456527,0.0776048246,1.5730013878
N,0,1.5319276346,-0.0868117821,-0.0820218871
H,0,1.4829631326,-0.3810054525,-1.0494984582
H,0,2.0105711093,0.8064744446,-0.065195657

Fluorocyclopropane
H G4 -217.00656
H W1BD -217.16525
C,0,-0.877747839,0.7599442502,-0.1270275851
C,0,0.2505353897,-0.0000077688,0.4739804373
C,0,-0.8777482098,-0.7599401027,-0.1270519076
H,0,-1.553817901,1.2890453323,0.5294970056
H,0,-0.6819029524,1.2460036093,-1.0718530497
H,0,-0.6819035377,-1.2459693117,-1.0718929164
H,0,-1.5538185259,-1.2890618427,0.5294557471
F,0,1.4488157222,0.0000027714,-0.2019973634

H,0,0.4058868538,-0.0000249373,1.5442036322

1,1,2-Trifluorocyclopropane
H G4 -415.45004
H W1BD -415.82602
C,0,-1.7292961928,-0.2467876524,0.0244329053
C,0,-0.2488304933,-0.2210868899,0.0148838013
C,0,-0.9687536241,1.0773739703,0.0233319807
H,0,-2.243894684,-0.5589634084,0.9238115715
H,0,-0.9778628937,1.6713572826,0.9260850165
H,0,-0.9693010681,1.6193941504,-0.9124795292
F,0,-2.3837684324,-0.6044660463,-1.115631655
F,0,0.4142606418,-0.5976699177,-1.0913997753
F,0,0.4178308865,-0.6224024185,1.1187745941

Chlorocyclopropane
H G4 -577.27233
H W1BD -578.74348
H,0,-0.3171179505,-1.4784595153,1.2506844167
C,0,0.5992006815,-1.1926996512,0.7558045545
C,0,0.5992006815,-1.1926996512,-0.7558045545
C,0,0.5972169075,0.0951245263,0.
H,0,1.5037258897,-1.4640358203,1.2822502361
H,0,-0.3171179505,-1.4784595153,-1.2506844167
H,0,1.5037258897,-1.4640358203,-1.2822502361
H,0,1.4852656463,0.7071141622,0.
Cl,0,-0.8591037969,1.1097482851,0.

**C1 epoxide radicals**
Oxiranyl radical
H G4 -153.03206
H W1BD -153.13069
C,0,-0.5927171589,-0.5640886903,-0.1528201169
C,0,0.7947558556,-0.1952237366,0.0190423287
O,0,-0.3254820511,0.7597110797,0.0319867875
H,0,-1.2931013111,-1.101863637,0.4775100422
H,0,1.4386512055,-0.1193225344,-0.8487203511
H,0,1.2941814599,-0.2900534813,0.9771303097

Methyloxirane C1 radical
H G4 -192.31964
H W1BD -192.43446
C,0,-1.1033508692,-0.5985440067,0.045457239
C,0,0.148262174,-0.0304093682,-0.4053375718
O,0,-0.7795419306,0.8358578078,0.1125696657
H,0,-1.8737855865,-0.8562286781,-0.6714889551
H,0,-1.1899952407,-1.0661209756,1.0223400863
C,0,1.5506796364,-0.1043276242,0.0691095086
H,0,2.0634667215,-0.9505105465,-0.3865899556
H,0,2.0903905577,0.8065796719,-0.1921503412
H,0,1.5920245375,-0.2198192805,1.160446324

Aminooxirane C1 radical, $NH_2$ lone pair rotated “in” towards ring
H G4 -208.37149
H W1BD -208.50657
C,0,-0.550473449,-2.0729765052,0.0293277501
C,0,0.8519076129,-2.4460890123,0.1418015518
O,0,0.5634210249,-1.1587333261,-0.2439354497
H,0,-1.1203212865,-2.3730906375,-0.8410139717
H,0,-1.1163544816,-1.804189947,0.9152988647
N,0,1.5798913667,-2.6571820111,1.3204077399
H,0,1.6959158726,-3.6408250779,1.5228919879
H,0,2.48547683,-2.2047117228,1.2913559569

Aminooxirane C1 radical, +2.4 kcal/mol, $NH_2$ lone pair rotated “out” from ring
H G4 -208.36769
H W1BD -208.50281
C,0,-0.5448820812,-2.1823261605,-0.0322122792
C,0,0.908732601,-2.2003908928,0.1508634655
O,0,0.2994748601,-0.9790767056,0.0403214156
H,0,-0.9687398604,-2.3834675567,-1.0079189975
H,0,-1.2152749191,-2.2843485535,0.8162417151
N,0,1.7361948136,-2.4426883395,1.2270310548
H,0,1.6314637755,-1.821005167,2.020944009
H,0,1.8603124805,-3.4101354144,1.4749196768

Aminooxirane C1 radical, +2.8 kcal/mol, $NH_2$ lone pair rotated *anti* to ring
H G4 -208.36713
H W1BD -208.50208
C,0,-0.4729961044,-2.1352080973,0.0197350187
C,0,0.9662114737,-2.143297506,0.0971251685
O,0,0.3449500161,-0.9357926914,-0.2171236401
H,0,-0.9772982278,-2.529465075,-0.8548640627
H,0,-1.0785594269,-2.0719428461,0.9207965381
N,0,1.8084860547,-2.3797552553,1.161351642
H,0,2.5673661623,-1.7210424663,1.249911787
H,0,1.3713070623,-2.5862478725,2.0524850885

Hydroxyoxirane C1 radical, OH rotated “out” from ring
H G4 -228.24364
H W1BD-228.40817
C,0,2.2440312226,-1.0356977329,-1.2588193653
C,0,1.8843776413,-0.4767605012,0.025054258
O,0,1.1002840137,-1.5011559462,-0.439115878
H,0,1.9708374122,-0.5222537609,-2.1715483925
H,0,3.0413364325,-1.7674955892,-1.3282405769
O,0,2.616534081,-0.5905261733,1.163015516
H,0,2.0197589367,-0.7698758963,1.9006092187

Hydroxyoxirane C1 radical, +0.6 kcal/mol, OH rotated *anti* to ring
H G4 -228.24262
H W1BD -228.40725

C,0,0.4435442645,-0.6860811738,-0.0785488897
C,0,1.8567756079,-0.3426262089,0.0788097073
O,0,0.9736477786,0.6771147477,0.1480681469
H,0,0.0339663994,-0.8589670373,-1.0651584167
H,0,-0.1269800403,-1.0594324477,0.7645798686
O,0,2.6609874741,-0.5404530508,1.1595930958
H,0,3.1964308458,-1.3247126793,0.9972692877

Fluorooxirane C1 radical
H G4 -252.26127
H W1BD -252.46816
C,0,-1.0141502028,-0.5995416328,0.0427850663
C,0,0.2160471733,-0.0010644793,-0.4369216615
O,0,-0.6554226265,0.8511644652,0.1227317231
H,0,-1.8099784906,-0.8311825875,-0.6515909674
H,0,-1.0471618343,-1.0737079064,1.0160552223
F,0,1.4259619808,-0.1464188592,0.1155656172

*Cis*-1,2-difluorooxirane C1 radical
H G4 -351.48807
H W1BD -351.80320
C,0,-0.867809947,1.0258091426,-0.0125612601
C,0,0.5774731724,1.0734612612,0.042174944
O,0,-0.1005427413,2.2570456088,0.0059668475
H,0,-1.4427980092,0.8331285819,-0.9084054711
F,0,1.3233395304,0.793201752,1.1062778422
F,0,-1.5828836852,0.8168294736,1.1038195175

*Trans*-1,2-difluorooxirane C1 radical
H G4 -351.48943
H W1BD -351.80453
C,0,-0.4612116181,1.4759243437,0.0194459193
C,0,0.9802873993,1.5195384194,0.0442061122
O,0,0.3158174649,2.7054535838,0.0377628851
H,0,-1.0955013475,1.3376627085,0.8865086321
F,0,1.7852406229,1.2323165958,1.0651791777
F,0,-1.1038877416,1.2101443687,-1.1255717265

Chlorooxirane C1 radical
H G4 -612.52485
H W1BD -614.04426
C,0,0.7869832271,-0.2578748239,-0.0055220837
C,0,2.2319327881,-0.1872435184,0.0782497652
O,0,1.5889424488,0.9925620731,0.0004593769
H,0,0.3163698178,-0.4875374758,-0.9525131904
H,0,0.1886429889,-0.3826764849,0.8887322687
Cl,0,3.2835531894,-0.5855785101,1.4020312734

**C1 cyclopropyl radicals**
Cyclopropyl radical
H G4 -117.11434

H W1BD -117.16443
C,0,-0.7665087744,-0.364236083,0.0327711314
C,0,0.0000003247,0.8665137075,-0.1616918183
C,0,0.7665085898,-0.3642366028,0.0327711288
H,0,-1.2958315082,-0.5178386688,0.9673972495
H,0,-1.2511012716,-0.8238091643,-0.8219980675
H,0,0.0000006462,1.8121733845,0.3551862023
H,0,1.2511007725,-0.8238100127,-0.8219980718
H,0,1.2958312227,-0.5178395475,0.9673972451

Methylcyclopropane C1 radical
H G4 -156.39900
H W1BD -156.46530
C,0,0.226805509,-0.7243009562,0.0052823989
C,0,1.7633935021,-0.7260363407,0.0111806833
C,0,0.997290318,0.5022758273,-0.2038886425
H,0,-0.2880479316,-1.1749444977,-0.8391584692
H,0,-0.2679470021,-0.8925644119,0.955790425
H,0,2.2836903389,-1.1778526575,-0.8292907884
C,0,1.0025259353,1.5265438361,-1.2682435464
H,0,0.1174744424,2.1631884653,-1.2186199202
H,0,1.8885514919,2.1612599937,-1.2117665686
H,0,1.0058684346,1.0558594917,-2.2634383994
H,0,2.2504493015,-0.8954038301,0.9654616176

Hydroxycyclopropane C1 radical
H G4 -192.32139
H W1BD -192.43728
H,0,-0.8 103805334,-1.2545295281,1.0378385947
C,0,-0.9049521878,-0.758919088,0.0780178434
C,0,-0.8764056408,0.7918813478,0.0786801889
C,0,0.2422722282,-0.0174382019,-0.4341482728
H,0,-1.5816790449,-1.2409964243,-0.6159631836
H,0,-0.7662321148,1.2803192222,1.0413145155
H,0,-1.5296242788,1.3041332349,-0.6159435581
O,0,1.4913866958,-0.1141435945,0.0998302307
H,0,1.9181578766,0.7486170319,0.0444416413

Aminocyclopropane C1 radical
H G4 -172.45420
H W1BD -172.54052
H,0,1.0592905571,-0.8157829993,-1.2626799464
C,0,0.098839852,-0.9227902034,-0.768817007
C,0,0.098839852,-0.9227902034,0.768817007
C,0,-0.4304253338,0.2140841722,0.
H,0,-0.590766476,-1.5916297227,-1.2687011349
H,0,1.0592905571,-0.8157829993,1.2626799464
H,0,-0.590766476,-1.5916297227,1.2687011349
N,0,0.0921469938,1.5005625972,0.
H,0,-0.1137337635,2.0344720405,-0.832446888
H,0,-0.1137337635,2.0344720405,0.832446888

Fluorocyclopropane C1 radical
H G4 -216.33748
H W1BD -216.49582
C,0,0.8623564221,0.7819203451,0.0786276643
C,0,-0.2520365293,0.0000003217,-0.4451705153
C,0,0.8623551966,-0.7819220363,0.0786268402
H,0,1.540146983,1.2723970828,-0.6075185947
H,0,0.7360234502,1.2789149394,1.0330345668
H,0,0.7360214098,-1.2789174739,1.0330331964
H,0,1.5401449636,-1.2723991488,-0.6075199685
F,0,-1.475357896,0.0000010139,0.1085568109

Chlorocyclopropane C1 radical
H G4 -576.60673
H W1BD -578.07744
H,0,1.0196828072,1.2534408178,1.2806806571
C,0,0.0668983025,1.352953787,0.7736255774
C,0,0.0668983025,1.352953787,-0.7736255774
C,0,-0.4441612961,0.2243028111,0.
H,0,-0.6399130253,2.0067498356,1.2709832872
H,0,1.0196828072,1.2534408178,-1.2806806571
H,0,-0.6399130253,2.0067498356,-1.2709832872
Cl,0,0.0639191285,-1.410017695,0.

**C1 epoxide radical inversion transition states**
Oxiranyl radical inversion TS
H G4 -153.02363
H W1BD -153.12233
C,0,0.5973446578,-0.5433219249,-0.0000584737
C,0,-0.792179655,-0.231507212,-0.0000276781
O,0,0.2989536764,0.7839040471,-0.0000292827
H,0,1.5059669081,-1.1050725904,-0.0000631172
H,0,-1.3716059837,-0.2478342634,0.9183013769
H,0,-1.3716386036,-0.2477940564,-0.9183368251

Methyloxirane C1 radical inversion TS
H G4 -192.30810
H W1BD -192.42290
C,0,-0.1668872965,-0.1116334798,0.0000286812
C,0,1.1757699785,-0.586117487,-0.0000489921
O,0,0.8013651307,0.8572401586,0.0002915406
H,0,1.6641982611,-0.9089690807,-0.9166145074
H,0,1.6641470839,-0.9094147274,0.9163866807
C,0,-1.6274777616,-0.0996155267,-0.0000235032
H,0,-2.0024978351,-1.1226305294,-0.0003713375
H,0,-2.0387017809,0.4089566174,0.8819057051
H,0,-2.0386197801,0.409529055,-0.8816592672

Aminooxirane C1 radical inversion TS, N lone pair *anti* to oxygen
H G4 -208.34984

H W1BD -208.48471
C,0,-0.4662118044,-2.2205790886,0.0377480362
C,0,0.9423322701,-2.3544869292,-0.089128046
O,0,0.4757600848,-1.0685228598,-0.239368321
H,0,-1.1397085894,-2.4328313049,-0.7898386568
H,0,-0.9511598101,-2.1542238248,1.0093297992
N,0,2.1460867981,-2.9921681805,-0.1165302801
H,0,2.623193471,-2.9813317917,-1.0128832528
H,0,2.79425701,-2.7285545305,0.6193908914

Aminooxirane C1 radical inversion TS, +0.9 kcal/mol, N lone pair *syn* to oxygen
H G4 -208.34831
H W1BD -208.48327
C,0,1.123203,0.610574,-0.000158
C,0,-0.204124,0.085062,0.000014
O,0,0.786312,-0.860653,0.000186
H,0,1.610364,0.940473,-0.91534
H,0,1.610395,0.940874,0.914859
N,0,-1.564807,0.019304,0.000097
H,0,-2.03601,0.34879,0.833855
H,0,-2.036072,0.346141,-0.834673

Hydroxyoxirane C1 radical inversion TS
H G4 -228.21223
H W1BD -228.37667
C,0,-1.1088223021,-0.595433253,-0.0230945092
C,0,0.218358016,-0.1063342113,0.0146208298
O,0,-0.7214349502,0.8831183145,0.0037642718
H,0,-1.5921684115,-0.8903729802,-0.9531464436
H,0,-1.627271275,-0.9143127985,0.8787603061
O,0,1.5453955024,-0.1360471696,0.102687387
H,0,1.9651234203,0.0247420982,-0.7597548418

Fluorooxirane C1 radical inversion TS
H G4 -252.22087
H W1BD -252.42763
C,0,0.2236208497,-0.1019928563,-0.0000100441
C,0,-1.0904299643,-0.603159083,0.0000346709
O,0,-0.6833624472,0.894026071,0.0000377316
H,0,-1.5935122989,-0.8982712523,0.918723723
H,0,-1.5935836538,-0.898248112,-0.9186227318
F,0,1.5378065145,-0.1256507674,-0.0000613497

1,2-Difluorooxirane C1 radical inversion TS
H G4 -351.45478
H W1BD -351.76978
C,0,-0.6245228045,-0.0211343968,0.1105230073
C,0,0.7175310221,0.0176866495,0.4661260056
O,0,-0.0424222551,1.1524184259,-0.229613053
H,0,1.1178270439,0.2962559078,1.4353223964
F,0,1.680327804,-0.557871111,-0.280471383

F,0,-1.8303459104,-0.4993967954,-0.0604237332

Chlorooxirane C1 radical inversion TS
H G4 -612.50373
H W1BD -614.02257
C,0,1.5477492225,0.6128654977,-0.0000049917
C,0,0.2326288625,0.0789493051,0.0000040532
O,0,1.2112110334,-0.862907973,-0.0000237888
H,0,2.0209951536,0.9450140748,-0.9196245644
H,0,2.0210196558,0.9449845932,0.9196125815
Cl,0,-1.4291169278,0.0520945022,0.0000247101

**C1 cyclopropyl radical inversion transition states**
Cyclopropyl radical inversion TS
H G4 -117.11203
H W1BD -117.16228
C,0,0.6464612262,-0.5314427742,-0.0000605573
C,0,-0.7641474642,-0.1963919556,-0.000047012
H,0,1.3986662908,-1.2952106072,-0.000087944
H,0,-1.3488130674,-0.3257685957,0.9074603999
H,0,-1.3488065833,-0.3256900565,-0.9075697707
C,0,0.3330004852,0.8841127094,-0.0000079344
H,0,0.4712474063,1.4666970886,0.9075303658
H,0,0.4712550964,1.4667718813,-0.9074971073

Methylcyclopropyl C1 radical inversion TS
H G4 -156.39452
H W1BD -156.46077
C,0,-1.0082543701,-0.7769685754,0.0000044361
C,0,-1.0248074475,0.7676267399,-0.0000072574
C,0,0.2099591116,0.009281264,0.000002022
H,0,-1.330280675,-1.2850534916,0.9074688275
H,0,-1.3302747674,-1.2850673297,-0.9074543192
H,0,-1.3586266123,1.268717802,0.9071645666
H,0,-1.3586209708,1.2687043251,-0.9071886389
C,0,1.6796971944,0.0064332999,-0.0000006701
H,0,2.0856153147,-0.5146248711,-0.8761107597
H,0,2.0797205735,1.0208114274,-0.0000392104
H,0,2.0856196489,-0.5145575904,0.8761480034

Hydroxycyclopropyl C1 radical inversion TS
H G4 -192.30575
H W1BD -192.42182
H,0,1.2568396367,1.3069186502,0.908985306
C,0,0.9161013025,0.8075742589,0.0000048535
C,0,0.953980794,-0.7750227909,0.0000080454
C,0,-0.2535933585,-0.0192497411,0.0000007865
H,0,1.2568488456,1.3069153628,-0.9089740326
H,0,1.3183126905,-1.2564863446,0.9090123955
H,0,1.3183217074,-1.2564892586,-0.9089911736
O,0,-1.6021214345,-0.1173103341,-0.0000066972

H,0,-1.9742661836,0.7718531975,-0.0000104835

Aminocyclopropyl C1 radical inversion TS
H G4 -172.44020
H W1BD -172.52671
H,0,1.3496558139,-1.2803855221,-0.877640884
C,0,0.9667545102,-0.7810388616,0.0142147139
C,0,0.9667547599,0.7810387819,0.0142146797
C,0,-0.236535974,0.0000001648,-0.0357095324
H,0,1.2789445478,-1.2745426904,0.9376178428
H,0,1.3496562284,1.2803852808,-0.8776409487
H,0,1.2789449507,1.2745425535,0.9376177812
N,0,-1.613729966,0.0000004038,-0.0790371049
H,0,-2.0456975685,-0.8344291425,0.2939528243
H,0,-2.0456973026,0.8344300911,0.2939526281

Fluorocyclopropyl C1 radical inversion TS
H G4 -216.31951
H W1BD -216.47795
C,0,0.,0.80140988,-0.9100019671
C,0,0.,-0.80140988,-0.9100019671
C,0,0.,0.,0.2649385892
H,0,0.9100777192,1.2884401787,-1.2599538182
H,0,-0.9100777192,1.2884401787,-1.2599538182
H,0,0.9100777192,-1.2884401787,-1.2599538182
H,0,-0.9100777192,-1.2884401787,-1.2599538182
F,0,0.,0.,1.5928446249

Chlorocyclopropyl C1 radical inversion TS
H G4 -576.59882
H W1BD failed after optimization
H,0,1.3443357208,1.2781305827,0.9113327908
C,0,1.010798693,0.7788108627,-0.0000244461
C,0,0.9983203728,-0.7910322021,-0.0000266588
C,0,-0.2047690381,0.003503041,0.0000081501
H,0,1.3442850187,1.278133586,-0.9113985877
H,0,1.3238743246,-1.295595136,0.9113294137
H,0,1.3238235589,-1.2955921844,-0.9114024962
Cl,0,-1.8894685806,0.0169127702,0.0000557542

**C1 epoxide radical ring opening transition states**
TS-A Oxiranyl radical ring opening TS
H G4 -153.00981
H W1BD -153.10776
C,0,0.9576268336,-0.0348086553,0.0290675971
H,0,1.6245678732,-0.1077064869,-0.8236878599
H,0,1.2169548203,-0.6040437247,0.9119868276
C,0,-0.2902186604,0.6467209731,-0.043746412
H,0,-0.7642920395,1.5508411238,0.3167360226
O,0,-0.7573278273,-0.5661352301,-0.0468761754

TS-B Oxiranyl radical ring opening TS
H G4 -153.00639
H W1BD -153.10606
C,0,-0.9419305626,-0.0771411713,-0.0074106062
H,0,-1.5979504966,-0.0282778019,0.8611738974
H,0,-1.2244913389,-0.7639742504,-0.7924591186
C,0,0.2435043649,0.6540244353,-0.133150165
H,0,0.6347017461,1.6177041636,0.155189557
O,0,0.792370287,-0.5370713754,0.0825034354

TS-A Methyloxirane C1 radical ring opening TS
H G4 -192.29782
H W1BD -192.41216
C,0,-1.1358146262,-0.7226665458,0.040333928
H,0,-1.7081632913,-1.1787108372,-0.7603955714
H,0,-1.5870295138,-0.7107327993,1.0253688184
C,0,0.0911129936,-0.033819692,-0.2025770782
O,0,-0.6757568858,1.0008859914,0.006085316
C,0,1.5420615764,-0.1695192017,0.0407460964
H,0,1.8975325719,-1.1415869509,-0.2995217319
H,0,2.0926852036,0.6115783346,-0.4848265558
H,0,1.7749609716,-0.0758532991,1.1105037784

TS-B Methyloxirane C1 radical ring opening TS
H G4 -192.29343
H W1BD -192.40915
C,0,1.1002785717,-0.7300618309,-0.005559134
H,0,1.2818925194,-1.3371107031,0.8834596902
H,0,1.9055801503,-0.6736032789,-0.7242640405
C,0,-0.0868211387,-0.0228719889,-0.2300996288
O,0,0.7055865822,1.0093919245,0.0673846802
C,0,-1.5372576955,-0.1501706125,0.0066441265
H,0,-2.062296834,0.7071720467,-0.4185399205
H,0,-1.9268643105,-1.0573157126,-0.4536901154
H,0,-1.7779388448,-0.1794388442,1.0792293424
TS-A Aminooxirane C1 radical ring opening TS, N lone pair rotated “in” towards ring
H G4 -208.35111
H W1BD -208.48591
C,0,-1.121483625,-0.6937286085,0.0563283792
H,0,-1.7879429786,-1.0391000541,-0.7201322784
H,0,-1.4709425494,-0.7014964745,1.0811252645
C,0,0.1264928447,-0.0351326546,-0.2808302137
O,0,-0.599612821,1.0008197686,0.0092548801
N,0,1.4373250587,-0.2058870884,0.1416989631
H,0,1.8860902468,-1.0229036597,-0.2467522288
H,0,1.9986268237,0.6238827711,0.0050812339

TS-B Aminooxirane C1 radical ring opening TS, N lone pair rotated “in” towards ring
H G4 -208.34429
H W1BD -208.47985
C,0,-1.0529026042,-0.73533674,-0.0387361972

H,0,-1.135382212,-1.3029519045,-0.9664089111
H,0,-1.8987547293,-0.7352558213,0.6307022253
C,0,0.1048278836,-0.0267520799,0.3051846755
O,0,-0.6504934825,1.0187797881,-0.0706869355
N,0,1.427323213,-0.2051175711,-0.1170576424
H,0,1.8772724241,0.6846649586,-0.2910012114
H,0,1.9791775073,-0.7509126298,0.5299819969

TS-A Aminooxirane C1 radical ring opening TS, +1.6 kcal/mol, N lone pair rotated “out” from ring
H G4 -208.34859
H W1BD -208.48338
C,0,-1.129966849,-0.6874806191,0.0800868082
H,0,-1.8168434988,-1.0143658746,-0.6863166139
H,0,-1.472296356,-0.6793355811,1.108630592
C,0,0.1295507619,-0.0508879429,-0.286189421
O,0,-0.5878954046,0.9896610365,0.0096576342
N,0,1.4633431521,-0.1779358449,0.0018342804
H,0,1.8390003961,0.5128664353,0.6413271207
H,0,1.8108877982,-1.1123786093,0.1366095994

TS-B Aminooxirane C1 radical ring opening TS, +1.8 kcal/mol, N lone pair rotated “out” from ring
H G4 -208.34138
H W1BD -208.47696
C,0,1.0428388443,-0.7477943717,0.0470204014
H,0,1.1385506988,-1.3232351267,0.9709893371
H,0,1.8816868734,-0.7625700826,-0.6324331246
C,0,-0.1106737274,-0.0255723642,-0.2897324537
O,0,0.664404161,1.0022817531,0.0830969474
N,0,-1.4627986644,-0.0998027308,-0.0186095375
H,0,-1.8803567327,-0.9958695488,-0.2112281619
H,0,-1.7530754531,0.2859254717,0.8756375917

TS-A Hydroxyoxirane C1 radical ring opening TS, OH rotated “out” from ring
H G4 -228.22401
H W1BD -228.38864
C,0,-1.1364089797,-0.6478064053,0.0667404285
H,0,-1.8485486201,-0.918905213,-0.6975265514
H,0,-1.4508475979,-0.6530743435,1.1020383257
C,0,0.1384290493,-0.0504010054,-0.2983055511
O,0,-0.4909797964,1.0239426923,0.0275951413
O,0,1.3973496054,-0.353873063,0.083188063
H,0,1.8539353395,0.4766253379,0.281926144

TS-B Hydroxyoxirane C1 radical ring opening TS, OH rotated “out” from ring
H G4 -228.21587
H W1BD -228.38114
C,0,1.0199324665,-0.7324792091,0.0256504537
H,0,1.0596861062,-1.3378767716,0.9305752418
H,0,1.8965345873,-0.6898221491,-0.6002156518

C,0,-0.1157677978,-0.0079011385,-0.3357269767
O,0,0.5878076611,1.0416813037,0.1170826391
O,0,-1.3919766616,-0.2983644786,0.0406236273
H,0,-1.8557633618,0.5349204432,0.1911716666

TS-A Hydroxyoxirane C1 radical ring opening TS, +1.0 kcal/mol, OH rotated *anti* to ring
H G4 -228.22227
H W1BD -228.38707
C,0,-1.0869939613,-0.7164394088,0.0571349802
H,0,-1.7638669109,-1.0333805322,-0.721625988
H,0,-1.4325735552,-0.7221782072,1.0826021661
C,0,0.1489781533,-0.0086490273,-0.2906474827
O,0,-0.5641934776,1.0032691982,0.0201775363
O,0,1.4342911023,-0.116184044,0.1224024856
H,0,1.7760796493,-0.9761589787,-0.1478366974

TS-B Hydroxyoxirane C1 radical ring opening TS, +1.2 kcal/mol, OH rotated *anti* to ring
H G4 -228.21379
H W1BD -228.37922
C,0,0.9887807609,-0.77875852,0.0238540436
H,0,1.0401428144,-1.367299716,0.9398869179
H,0,1.8370507122,-0.8011016813,-0.6418605226
C,0,-0.1229587169,0.0107384681,-0.3025164546
O,0,0.6484739029,1.0167979221,0.0933789595
O,0,-1.4121213331,-0.0895029336,0.1353143337
H,0,-1.8793931404,-0.7151615393,-0.4271062775

TS-A Fluorooxirane C1 radical ring opening TS
H G4 -252.24088
H W1BD -252.44832
C,0,-1.0928425497,-0.6879057035,0.0465054336
H,0,-1.796641067,-0.9610459847,-0.7248455431
H,0,-1.4094933253,-0.705847843,1.0800010679
C,0,0.1485662129,-0.0087206227,-0.3086262235
O,0,-0.4735304544,1.0340305961,0.0415526604
F,0,1.3968201836,-0.2676314422,0.1002116046

TS-B Fluorooxirane C1 radical ring opening TS
H G4 -252.23310
H W1BD -252.44097
C,0,0.9721786533,-0.7678223266,0.013255965
H,0,0.9826283029,-1.3855526458,0.9092428938
H,0,1.8536704192,-0.7435971818,-0.6070290792
C,0,-0.1220256141,0.0247471539,-0.3363705633
O,0,0.5750313483,1.0458998146,0.1300298036
F,0,-1.3830881096,-0.2035208143,0.0719389801

TS-A *Cis*-1,2-difluorooxirane radical ring opening TS
H G4 -351.45730
H W1BD -351.77408
C,0,0.7602669118,-0.1121020832,0.5778114957

H,0,1.2656307095,0.1822543719,1.4865838504
C,0,-0.6466950177,0.1494486929,0.3424584544
O,0,-0.2668929796,1.1991900182,-0.2615571734
F,0,-1.5765936291,-0.6767359968,-0.1484463682
F,0,1.5944510051,-0.4397500029,-0.3995342589

TS-B *Cis*-1,2-difluorooxirane radical ring opening TS
H G4 –351.46408
H W1BD -351.77982
C,0,-0.7630537611,0.2444688328,0.4214628413
H,0,-1.2691822061,0.9832359403,1.0243653936
C,0,0.631189563,0.1363661676,0.3661874252
O,0,0.579229032,1.2393912207,-0.3570768328
F,0,1.2913556514,-0.9574592488,-0.0450657392
F,0,-1.5743942792,-0.5089379126,-0.2810960882

TS-A *Trans*-1,2-difluorooxirane radical ring opening TS
H G4 -351.46734
H W1BD -351.78297
C,0,-1.0817766123,-0.6556384742,0.0506246363
C,0,0.1393182427,0.0086866556,-0.3015808757
O,0,-0.4522366113,1.0566284492,0.1249226803
F,0,1.4030860775,-0.3000380169,0.0102082317
H,0,-1.5166962087,-0.6709610967,1.0413359959
F,0,-1.8913883179,-1.104117517,-0.8823254886

TS-B *Trans*-1,2-difluorooxirane radical ring opening TS
H G4 -351.45677
H W1BD -351.77304
C,0,0.6531664468,-0.3856820523,0.3413875676
H,0,0.734269695,-0.8235463278,1.3348141236
C,0,-0.5271574137,0.0347164959,-0.2827002508
O,0,-0.285038118,1.2551211139,0.1213416806
F,0,-1.7135873985,-0.566278536,-0.0500390714
F,0,1.8095447884,-0.2297016937,-0.2393980495

TS-A Chlorooxirane C1 radical ring opening TS
H G4 -612.50236
H W1BD -614.02239
C,0,0.2668455148,0.1147338381,0.5054618068
H,0,0.1447739099,-0.2713352224,1.5075715456
C,0,-0.8860582328,0.6098689709,-0.2139232926
O,0,-1.2586308427,-0.605540378,-0.1857646109
H,0,1.2056221702,-0.0444956234,-0.006903069
Cl,0,-1.9079506494,1.9998906549,0.0328639501

TS-B Chlorooxirane C1 radical ring opening TS
H G4 -612.49681
H W1BD -614.01766
C,0,1.3800785326,-0.8217920665,0.0206272958
H,0,1.3476334624,-1.4615808325,0.9001884796

H,0,2.268153343,-0.8420267712,-0.5924966378
C,0,0.3446890014,0.0506686478,-0.3256063713
O,0,1.1386565638,0.9977746556,0.1404865113
Cl,0,-1.3495279032,-0.0667196333,0.0229667224

**C1 cyclopropyl radical ring opening transition states**
Cyclopropyl radical ring opening TS
H G4 -117.07804
H W1BD -117.12846
C,0,-1.0397849737,-0.205587109,0.0652215393
H,0,-1.4613157048,-0.2434481575,1.0603459928
H,0,-1.4276905163,-0.8973864698,-0.6701246203
C,0,0.0783934262,0.6933120956,-0.2263022752
H,0,0.1805425069,1.6868001519,0.1853364023
C,0,0.9649048279,-0.3322590804,0.028892579
H,0,1.7701178993,-0.2683445174,0.7618780388
H,0,0.9289205343,-1.2554449133,-0.5354446566

Methylcyclopropane C1 radical ring opening TS
H G4 -156.36206
H W1BD -156.42871
C,0,0.938991317,-0.9745264152,0.0580505519
H,0,0.9563779855,-1.3810814884,1.0620895391
H,0,1.735568752,-1.2658212424,-0.6128294813
C,0,-0.0776031605,0.0146207164,-0.3042528959
C,0,0.7940516314,1.0277802476,0.040529535
H,0,0.5511899332,1.8017585772,0.7736509307
H,0,1.75667291,1.1355209765,-0.4416811446
C,0,-1.5286767661,-0.0671331897,0.010735927
H,0,-2.0370142881,0.8672072501,-0.2296658361
H,0,-2.0047137823,-0.8724698268,-0.5521802968
H,0,-1.7060695322,-0.2791246054,1.0746351709

Fluorocyclopropane C1 radical ring opening TS
H G4 -216.30287
H W1BD -216.46209
C,0,0.9035497936,-0.9505047033,0.0771505226
H,0,0.9566709317,-1.2584514284,1.113491598
H,0,1.6798065556,-1.2739048855,-0.5995133953
C,0,-0.1262560804,-0.0086249442,-0.3366388673
C,0,0.6208690124,1.0859504411,0.0351766864
H,0,0.3177968348,1.755719039,0.8394375538
H,0,1.5134746459,1.3417386763,-0.5147037933
F,0,-1.4081706936,-0.1410461948,0.069810695

**C2 epoxide radicals**
Methyloxirane C2 radical, radical *syn* to methyl
H G4 -192.31753
H W1BD -192.43242
C,0,1.0219061358,-0.601441128,-0.2746710544
C,0,-0.1382451513,-0.1431051969,0.4568874383

O,0,0.9229871411,0.7447359691,-0.0743034148
H,0,1.9084586333,-1.162449605,0.0027595101
H,0,-0.1226993335,-0.1709403598,1.5434116507
C,0,-1.4926790569,-0.0243621341,-0.175045456
H,0,-2.0942584044,-0.9003300437,0.0774921271
H,0,-2.0177793505,0.8597907631,0.1896871004
H,0,-1.4074376137,0.0354937352,-1.2584939014

Methyloxirane C2 radical, radical *anti* to methyl
H G4 -192.31732
H W1BD -192.42334
C,0,-1.0590961395,0.6461981289,0.0904507121
C,0,0.1409635857,-0.0468407306,0.5053974139
O,0,-0.9499300038,-0.6630859516,-0.283210866
H,0,-1.2339976696,1.4340496901,-0.6359719741
H,0,0.1704182271,-0.419228619,1.524581894
C,0,1.4643632572,0.0663243936,-0.1913908467
H,0,2.0984837698,0.7859959948,0.3307950398
H,0,1.9854628451,-0.892963764,-0.1979454375
H,0,1.335755128,0.4008858578,-1.2198639356

Aminooxirane C2 radical, radical *syn* to $NH_2$, N lone pair rotated *anti* to oxygen
H G4 -208.37136
H W1BD -208.50606
C,0,-1.1932489437,-0.3058734519,-0.0437706979
C,0,0.2340151212,-0.4449704746,-0.0066205181
O,0,-0.5692500335,0.8841313795,-0.2242198185
H,0,-1.9769716326,-0.6776082071,-0.6959999141
H,0,0.8024504995,-0.6502232497,-0.905506201
N,0,0.9268372578,-0.6621565631,1.1708729116
H,0,1.8353641653,-0.224417792,1.2184711478
H,0,0.382162976,-0.4960841012,2.0040957001

Aminooxirane C2 radical, radical *anti* to $NH_2$, N lone pair rotated *anti* to oxygen
H G4 -208.37060
H W1BD -208.50542
C,0,-1.2242815674,-0.4101295487,0.0655842005
C,0,0.2089101452,-0.4704792719,0.0553071602
O,0,-0.6457841938,0.8157195794,-0.0799334875
H,0,0.7189774372,-0.6782028002,-0.8762810226
N,0,0.9964308166,-0.6440964757,1.1833728556
H,0,1.9117391385,-0.2202721732,1.1291634477
H,0,0.5370830735,-0.4216334548,2.0538153702
H,0,-1.9558222498,-0.621517885,0.8406667559

Hydroxyoxirane C2 radical, radical *syn* to OH, OH rotated “in” towards ring
H G4 -228.24454
H W1BD -228.40918
C,0,-0.1076271483,-0.608541244,-0.0736712002
C,0,1.3246022149,-0.5652983355,-0.1108281481
O,0,0.4535038094,0.6139655221,0.1815530528

H,0,-0.9096864512,-0.7604995026,-0.7868406651
H,0,1.9010210526,-0.4263314475,-1.0160375765
O,0,2.1101876206,-1.0466762627,0.8914721767
H,0,1.5433628121,-1.3580070099,1.6077246302

Hydroxyoxirane C2 radical, radical *syn* to OH, +2.0 kcal/mol, OH rotated "out" from ring
H G4 -228.24151
H W1BD -228.40600
C,0,-0.1893818546,-0.4490587406,-0.07117331
C,0,1.2278370602,-0.6476866447,-0.0430875839
O,0,0.5727057772,0.6856332856,0.0030099083
H,0,-0.9687157823,-0.5942516658,-0.8101300138
H,0,1.8226382951,-0.7599464009,-0.944925054
O,0,1.8074372767,-1.108947085,1.1046984388
H,0,2.7427472077,-0.8777708886,1.1055848545

Hydroxyoxirane C2 radical, radical *anti* to OH, OH rotated "in" towards ring
H G4 -228.24340
H W1BD -228.40807
C,0,-1.0356399902,-0.1474420475,0.1430982967
C,0,0.389134267,-0.2433138333,-0.001697711
O,0,-0.3205660873,1.011537799,0.3467684626
H,0,-1.7360145304,-0.5280921444,0.879781543
H,0,0.8617868204,-0.2172451309,-0.9738538185
O,0,1.252904521,-0.7410953855,0.9313142271
H,0,0.7891085394,-0.8688358273,1.7669297002

Hydroxyoxirane C2 radical, radical *anti* to OH, +0.8 kcal/mol, OH rotated "out" from ring
H G4 -228.24234
H W1BD -228.40682
C,0,-1.0832863654,-0.0708813373,0.1383727314
C,0,0.3303984506,-0.2947282024,0.0865416125
O,0,-0.2886872751,1.0487536498,0.1757190881
H,0,-1.8189911722,-0.2781046053,0.9070531439
H,0,0.832960838,-0.4568316939,-0.8611247784
O,0,1.0047033496,-0.7202982382,1.196921244
H,0,1.9488584046,-0.5643180728,1.0835932785

Fluorooxirane C2 radical, radical *syn* to F
H G4 -252.26352
H W1BD -252.47052
C,0,0.9483669311,-0.622123574,-0.219529846
C,0,-0.2095273445,-0.0970929188,0.4319694296
O,0,0.8242892872,0.7476794651,-0.0919989524
H,0,1.8324628692,-1.1666510616,0.0870514158
H,0,-0.369492666,-0.019817642,1.5006108321
F,0,-1.3851770769,-0.0553442686,-0.2324168791

Fluorooxirane C2 radical, radical *anti* to F
H G4 -252.26407
H W1BD -252.47104

C,0,-0.9825354709,0.645725861,0.1201672138
C,0,0.2143787491,-0.0568278969,0.4651504738
O,0,-0.8496109959,-0.6785531332,-0.2642973059
H,0,-1.1949230102,1.4293115044,-0.596025911
H,0,0.4277514081,-0.4634863437,1.4451317337
F,0,1.3512813198,0.1033000083,-0.2518652044

1,1-Difluorooxirane C2 radical
H G4 -351.49755
H W1BD -351.81274
C,0,0.1072327495,0.160702686,-0.0123554074
C,0,0.2522897442,-1.2499877154,-0.0772796804
O,0,-1.0265209187,-0.6656349254,-0.0370702335
H,0,0.539688356,-1.9578652151,-0.8428788889
F,0,0.3081319071,0.8604856591,1.1016384483
F,0,0.2330114119,0.9800000009,-1.0585028182

Trifluorooxirane C2 radical
H G4 -450.72038
H W1BD -451.14367
C,0,-0.8998406843,0.253250595,0.0455053242
C,0,0.5402718645,0.2263975594,0.0052833129
O,0,-0.1878249297,1.4432371279,0.0265318286
F,0,1.2282085092,-0.0315659496,-1.0950325891
F,0,1.3005690673,0.0136029621,1.0706435916
F,0,-1.6805860571,-0.0076953448,1.0794702419

Chlorooxirane C2 radical, radical *syn* to Cl
H G4 -612.52068
H W1BD -614.04005
C,0,-1.0341951258,0.1127440843,0.3421387004
C,0,0.3963372903,0.0844534096,0.3914586968
O,0,-0.3648696565,1.3127793167,0.3527028426
H,0,-1.7738127649,-0.149625821,-0.4047336558
H,0,1.0401572878,-0.072163012,-0.4628753753
Cl,0,1.2432250291,-0.2938089675,1.8918061412

Chlorooxirane C2 radical, radical *anti* to Cl
H G4 -612.52202
H W1BD -614.04141
C,0,-1.0317878309,0.0798492692,0.480660994
C,0,0.399099861,0.0674511921,0.4208414412
O,0,-0.3717920645,1.2888462235,0.4362802654
H,0,0.9489678404,-0.1309921015,-0.4874950602
Cl,0,1.4019013278,-0.2191681563,1.8461435397
H,0,-1.7336538838,-0.1650169871,1.26843884

**C2 cyclopropyl radicals**
Methylcyclopropane C2 radical, radical *syn* to methyl
H G4 -156.39447
H W1BD -156.46061

C,0,1.017865292,0.7016748428,-0.0379249127
C,0,-0.1554590683,-0.1529867967,0.4549180973
C,0,0.9419387155,-0.7339381252,-0.3213811236
H,0,0.7976951497,1.4044858339,-0.8357605722
H,0,1.7535259313,1.067133529,0.6714349884
H,0,-0.1696305713,-0.3819622543,1.5181738058
H,0,1.6234607554,-1.5504369837,-0.1441591372
C,0,-1.5318486564,0.0489997996,-0.1456835582
H,0,-2.1364436166,-0.8543480001,-0.0441453078
H,0,-2.0598512378,0.8665865482,0.3487594734
H,0,-1.4608146935,0.2824446065,-1.208534753

Methylcyclopropane C2 radical, radical *anti* to methyl
H G4 -156.39479
H W1BD -156.46096
C,0,1.0911317639,-0.6069074598,-0.1847547141
C,0,0.9205050639,0.8354301142,0.0128857579
C,0,-0.1573092617,-0.0280312465,0.4966478467
H,0,1.0036904099,-1.0148066689,-1.1873920832
H,0,1.7841824355,-1.1516352445,0.4476663334
H,0,0.9185440882,1.6547499975,-0.6890863388
H,0,-0.1898469956,-0.206884052,1.5684569406
C,0,-1.5121341622,-0.0537688668,-0.1760316159
H,0,-2.0203970085,-1.00477771,-0.0018864462
H,0,-2.154993091,0.7427039788,0.2043938893
H,0,-1.4163712422,0.0827531581,-1.2543025697

Fluorocyclopropane C2 radical, radical *syn* to F
H G4 -216.33391
H W1BD -216.49223
C,0,-0.9490708075,-0.6941574159,-0.0412646053
C,0,0.2324009034,0.1189001304,0.4390978097
C,0,-0.8636066266,0.7533014005,-0.26737182
H,0,-0.7569285676,-1.3467639544,-0.8892386227
H,0,-1.6499716313,-1.1058927824,0.67980081
H,0,0.3974490364,0.2822815555,1.4980004105
H,0,-1.4944725766,1.6181275591,-0.1539273302
F,0,1.4366152697,-0.0571514928,-0.2132246519

Fluorocyclopropane C2 radical, radical *anti* to F
H G4 -216.33567
H W1BD -216.49401
C,0,1.0217622472,-0.5996754752,-0.1702455922
C,0,-0.2354628021,-0.0337505319,0.4700838273
C,0,0.830381071,0.8449380024,0.0405354203
H,0,1.6756629972,-1.1731652337,0.4797731799
H,0,0.9661100531,-0.9765066809,-1.1868311984
H,0,0.8413345267,1.6701830556,-0.6514598002
F,0,-1.4103511172,-0.0612414091,-0.2498756378
H,0,-0.4209859759,-0.2423997271,1.5170678011

1,1,2-Trifluorocyclopropane C2 radical
H G4 -414.78142
H W1BD -415.15592
C,0,-1.698287018,-0.2230514219,-0.0351729127
C,0,-0.2433240388,-0.2493406626,0.0104824359
C,0,-0.9919484057,1.0824778362,0.0031931615
H,0,-0.9875612775,1.6445007667,0.9304790566
H,0,-0.9432255831,1.6700493158,-0.9079760606
F,0,-2.4457687058,-0.6413158815,-1.0522704065
F,0,0.4661412488,-0.5884679488,-1.0807459143
F,0,0.4202360001,-0.6264539738,1.1171490201

**C2 epoxide radical inversion transition states**
Methyloxirane C2 radical inversion TS
H G4 -192.30952
H W1BD -192.42444
C,0,1.0328083031,0.6227087977,-0.0780771083
C,0,-0.1506710215,0.0664678262,0.489761613
O,0,0.9383524476,-0.7322299912,-0.165228477
H,0,1.7557200401,1.3382234279,-0.4073122483
H,0,-0.1809924956,-0.1123532175,1.5626418942
C,0,-1.4835140622,0.0519006667,-0.2003417288
H,0,-2.0279676854,-0.8669106876,0.0248979095
H,0,-2.0872852601,0.8920381228,0.1501750948
H,0,-1.3596382659,0.140172055,-1.278108949

Aminooxirane C2 radical inversion TS
H G4 -208.36514 G4 geometry given here
H W1BD failed with NIMAG=2, inversion and ring opening modes
C,0,0.5956090809,-0.509481377,0.0060529289
C,0,-0.7898563095,-0.2223503989,-0.0432257377
O,0,0.3988332666,0.822682657,0.1403847989
H,0,1.4708128996,-1.1269332029,-0.0538929583
H,0,-1.3931803106,-0.2178066132,0.8635182452
N,0,-1.5440029066,-0.2292347252,-1.2054501144
H,0,-2.3124232585,0.4294815201,-1.2177243412
H,0,-0.9929499619,-0.1837683098,-2.0519046015

Hydroxyoxirane C2 radical inversion TS, OH rotated “in” towards ring
H G4 -228.23821
H W1BD -228.40287
C,0,0.9445522688,0.6355148655,0.026091022
C,0,-0.2328272688,-0.0057567307,0.472598832
O,0,0.9205273356,-0.7025488749,-0.2366874641
H,0,1.6232303777,1.423827044,-0.2281590962
H,0,-0.3605947017,-0.3694133767,1.4843901144
O,0,-1.4357041664,0.0318164385,-0.164463745
H,0,-1.3301158452,0.4997526343,-1.0019306631

Hydroxyoxirane C2 radical inversion TS, +1.8 kcal/mol, OH rotated “out” from ring
H G4 -228.23544

H W1BD -228.39994
O,0,-0.916886396,-0.7333331351,-0.1814633531
C,0,0.2282251531,0.0461851402,0.4420758641
H,0,0.367914374,-0.2422810071,1.4807602403
C,0,-0.9713856153,0.6187725787,-0.0233323594
H,0,-1.6937681393,1.3692242727,-0.2659494394
O,0,1.3778548157,0.1500608067,-0.2857010084
H,0,2.0185578079,-0.4960556562,0.0333840559

Fluorooxirane C2 radical inversion TS
H G4 -252.25731
H W1BD -252.46434
C,0,-0.9572086309,0.6273546195,-0.042792793
C,0,0.2226172242,0.0439285751,0.4507031561
O,0,-0.8426331871,-0.7389407963,-0.1595542324
H,0,-1.7042897945,1.3368011845,-0.3265121955
H,0,0.4310152211,-0.1887496308,1.4884562473
F,0,1.3754001672,0.082651048,-0.2586841825

1,1-Difluorooxirane C2 radical inversion TS
H G4 -351.49149
H W1BD -351.80674
C,0,0.1113640269,0.1159981056,0.
C,0,0.5104056064,-1.2229061074,0.
O,0,-0.8549176447,-0.9308586561,0.
H,0,1.0715417727,-2.1327422098,0.
F,0,0.1126332227,0.9005381146,1.0783720461
F,0,0.1126332227,0.9005381146,-1.0783720461

Trifluorooxirane C2 radical inversion TS
H G4 -450.68809
H W1BD -451.11130
C,0,0.8602558424,0.0000001323,-0.0555969651
C,0,-0.5253971566,0.0000001291,-0.0752165294
O,0,0.2734363537,-0.0000018102,1.1832679281
F,0,2.1031037213,0.0000007769,-0.4470037496
F,0,-1.2849298447,-1.072974487,-0.2593980162
F,0,-1.2849299161,1.0729752725,-0.2593946679

Chlorooxirane C2 radical inversion TS
H G4 -612.51406
H W1BD -614.03356
C,0,-1.3826575156,0.6443230944,-0.0884192519
C,0,-0.3024436877,0.0117818323,0.5474521888
O,0,-1.3098876424,-0.7144180685,-0.2411065962
H,0,-2.0590382738,1.4045621193,-0.4150789455
H,0,-0.2728374204,-0.2514644551,1.596117242
Cl,0,1.34373854,0.0382034777,-0.1165416372

**C2 cyclopropyl radical inversion transition states**
Methylcyclopropyl C2 radical inversion TS

H G4 -156.39231
H W1BD -156.45859
C,0,0.9350189901,0.777504577,-0.147670335
C,0,1.0538093689,-0.6696337248,-0.1095587487
H,0,1.3651337819,1.7130945322,-0.4487680367
H,0,1.7557531941,-1.1376401368,0.5768174406
H,0,0.9014642614,-1.2429733435,-1.0217918016
C,0,-0.1622526691,0.0673011751,0.4828565689
H,0,-0.2020390777,0.0844414968,1.5717988015
C,0,-1.5271477827,-0.0450304846,-0.1697146814
H,0,-2.0568593018,-0.9333462144,0.1808110457
H,0,-2.1433655902,0.8262056256,0.0610036332
H,0,-1.435029175,-0.1091805026,-1.2546818867

Fluorocyclopropyl C2 radical inversion TS
H G4 -216.33375
H W1BD -216.49221
C,0,0.8529073793,0.7837143687,-0.142180189
C,0,0.9770228804,-0.6691894131,-0.091360045
H,0,1.3147261678,1.7259915799,-0.3668559816
H,0,1.6527629779,-1.1512557036,0.6127878753
H,0,0.8446502982,-1.2280184028,-1.016104855
C,0,-0.2368414825,0.0613431571,0.4550180395
H,0,-0.4257769772,0.0752949084,1.5232066963
F,0,-1.4319582439,-0.0542204947,-0.2346905405

1,1,2-Trifluorocyclopropyl C2 radical inversion transition state
H G4 -414.77109
H W1BD -415.14563
C,0,0.8865797506,-0.0293905682,-0.0000444346
C,0,0.2541770427,-1.3300201432,-0.002872105
H,0,0.2141819884,-1.9267456395,0.9101428208
H,0,0.2142083317,-1.9227835803,-0.9184652669
C,0,-0.5245294935,0.0165067548,0.0000354875
F,0,-1.249998488,0.3597566504,-1.0916986141
F,0,-1.2500266583,0.3550190805,1.0932306589
F,0,2.0413775264,0.6047604455,0.0013404534

**C2 epoxide radical ring opening transition states**
TS-A Methyloxirane radical ring opening TS, C2 *syn* radical to *trans* vinoxy
H G4 -192.29679
H W1BD -192.41107
C,0,0.2727188698,0.09280582,0.496985845
H,0,0.1762952652,-0.3424765845,1.4853955478
C,0,-0.899718691,0.6145850164,-0.1250439596
H,0,-1.5373256114,1.4893453916,-0.0799919385
O,0,-1.2488329342,-0.640762737,-0.1497084909
C,0,1.5835893406,-0.0310607984,-0.1950786664
H,0,2.3953650303,-0.1875347974,0.5168320012
H,0,1.5650726989,-0.898357543,-0.8617891227
H,0,1.7860490318,0.8451012323,-0.8131952159

TS-B Methyloxirane radical ring opening TS, C2 *syn* radical to *cis* vinoxy
H G4 -192.29099
H W1BD -192.40660
C,0,-0.2545757286,-0.5242308814,-0.2993250901
H,0,-0.3705765628,-1.12662263,-1.2045773859
C,0,0.9856384321,-0.4577798255,0.3539934358
H,0,1.8155422411,-1.1408304456,0.4861619105
O,0,1.0639089282,0.7583596651,-0.1791120419
C,0,-1.4815456288,0.1805204416,0.1586294354
H,0,-1.9244235667,0.7783522596,-0.6403798905
H,0,-2.2245275021,-0.5770584363,0.4353652982
H,0,-1.2800226124,0.8082958525,1.0221123285

TS-A Methyloxirane radical ring opening TS, C2 *anti* radical to *cis* vinoxy
H G4 -192.29196
H W1BD -192.40618
C,0,-0.2650659872,-0.5198912761,-0.3385252504
H,0,-0.4029306313,-1.0547483791,-1.2777385962
C,0,1.0284293743,-0.4909230221,0.2564872859
H,0,1.5705307418,-0.9302699984,1.0843592449
O,0,1.0573622506,0.7371425658,-0.1783015936
C,0,-1.4696774207,0.1873562871,0.1884004052
H,0,-1.9138650336,0.8531232664,-0.5558006452
H,0,-2.2292639713,-0.5649074236,0.4318337614
H,0,-1.2414503226,0.75352398,1.0879823881

TS-B Methyloxirane radical ring opening TS, C2 *anti* radical to *trans* vinoxy
H G4 -192.29500
H W1BD -192.41026
C,0,0.2654273013,-0.04321103,0.5119875688
H,0,0.1789074142,-0.6913859406,1.3743080082
C,0,-0.8924609525,0.6468145291,0.1162851656
H,0,-1.0841890293,1.5759854958,-0.4059604113
O,0,-1.2801471868,-0.5644450982,-0.2774515687
C,0,1.5685034294,-0.0100163616,-0.2083803691
H,0,2.3940519379,-0.3294788237,0.4282010196
H,0,1.5142502018,-0.7064506712,-1.0506564005
H,0,1.7719878839,0.9795709004,-0.6192920126

TS-A Aminooxirane radical ring opening TS, C2 *syn* radical to *trans* vinoxy
H G4 -208.36730
H W1BD -208.50200
C,0,0.3397621186,0.0583662373,0.4723769
H,0,0.3191242562,-0.4352931249,1.4368764284
C,0,-0.8467862939,0.6480931563,-0.0825850598
H,0,-1.4891042387,1.4547853648,0.2628806674
O,0,-1.2096572506,-0.6108660477,-0.2426170542
N,0,1.5047437767,0.0032540386,-0.1828361282
H,0,2.1656679592,-0.7307804207,0.0115341728
H,0,1.5602066725,0.4387697962,-1.0891039264

TS-B Aminooxirane radical ring opening TS, C2 *syn* radical to *cis* vinoxy
H G4 -208.36266
H W1BD -208.49603
C,0,-0.404252919,0.4662504162,0.2994072876
H,0,-0.5600760845,0.9088061844,1.2850487968
C,0,0.8750958794,0.4513967556,-0.3600207952
H,0,1.5189353702,1.3158751343,-0.5210078818
O,0,1.2733967194,-0.6637040265,0.2123522319
N,0,-1.4470936519,-0.1900640267,-0.1732512643
H,0,-1.3531220512,-0.6384714501,-1.073841911
H,0,-2.1917662624,-0.4997559871,0.432865536

TS-A Aminooxirane radical ring opening TS, C2 *anti* radical to *cis* vinoxy
H G4 -208.35767
H W1BD failed; either NIMAG=2 or optimized to TS-B *anti* radical to *trans* vinoxy
C,0,0.9332722335,-0.0901807392,0.0693268978
H,0,1.6421038398,-0.1436210221,-0.7598440098
C,0,-0.3280086905,0.6071265657,-0.0682127677
H,0,-0.718664017,1.4697919747,0.4722316725
O,0,-0.9699192576,-0.4772600682,-0.4049219045
N,0,1.2720472969,-0.8577366569,1.1087607587
H,0,0.6434193017,-0.8935885343,1.8986500374
H,0,1.8789668532,-1.6591619496,0.9866341455

TS-B Aminooxirane radical ring opening TS, C2 *anti* radical to *trans* vinoxy
H G4 -208.36739
H W1BD -208.50169
C,0,0.3174479679,-0.1002234301,0.4808733951
H,0,0.3311124669,-0.8269692576,1.2807460256
C,0,-0.885437582,0.6223177452,0.2294064618
H,0,-1.0269527318,1.6032512974,-0.2214140961
O,0,-1.1940130934,-0.5313466878,-0.3728280003
N,0,1.4757109741,0.0507527906,-0.1851097471
H,0,2.1396117756,-0.7061739224,-0.2141737393
H,0,1.5110342228,0.6974534649,-0.9550712998

TS-A Hydroxyoxirane radical ring opening TS, C2 *syn* radical to *trans* vinoxy, OH rotated “in” towards ring
H G4 -228.23783
H W1BD -228.39705
C,0,0.3611663564,-0.0379920537,0.461150257
H,0,0.3528023316,-0.6934311944,1.3215720411
C,0,-0.7897992943,0.6495380136,-0.0033179465
O,0,-1.2549410325,-0.5522037097,-0.2523871375
O,0,1.5199262482,-0.0811858659,-0.1834744896
H,0,1.4896324676,0.5567206284,-0.9130624685
H,0,-1.3437890769,1.5403711818,0.268862744

TS-B Hydroxyoxirane radical ring opening TS, C2 *syn* radical to *cis* vinoxy, OH rotated “in” towards ring

H G4 -228.22690
H W1BD -228.38900
C,0,-0.3998409926,0.480659448,0.284333138
H,0,-0.621803866,1.002752392,1.2131445647
C,0,0.8546537844,0.4715377007,-0.3685322031
H,0,1.5638614055,1.2797052976,-0.5230130664
O,0,1.1391995,-0.6806219792,0.2251094174
O,0,-1.4349656429,-0.2330641437,-0.10790041
H,0,-1.2090391884,-0.6586507154,-0.9525164406

TS-A Hydroxyoxirane radical ring opening TS, C2 *syn* radical to *trans* vinoxy, +2.4 kcal/mol, OH rotated “out” from ring
H G4 -228.23352
H W1BD -228.39315
C,0,0.344628013,0.0315774507,0.4238253968
H,0,0.3661772081,-0.5450846976,1.3430429708
C,0,-0.8374863265,0.6384869207,-0.05142448
O,0,-1.2294375009,-0.6100997115,-0.1894225007
O,0,1.4751077688,0.0569884179,-0.2833785083
H,0,2.095807692,-0.6042885353,0.0503009657
H,0,-1.4293028545,1.525065155,0.1293501556

TS-B Hydroxyoxirane radical ring opening TS, C2 *syn* radical to *cis* vinoxy, +4.7 kcal/mol, OH rotated “out” from ring
H G4 -228.21689
H W1BD -228.38146
C,0,-0.3832871849,0.4381750595,0.3037970448
H,0,-0.6159373968,0.8346444666,1.295696642
C,0,0.8886501381,0.5077636237,-0.2918675911
H,0,1.6024075467,1.3156540053,-0.4007150315
O,0,1.1431571791,-0.7115631347,0.1465024652
O,0,-1.3828193,-0.1975959229,-0.2938821203
H,0,-2.0497389821,-0.4682120975,0.3522115909

TS-A Hydroxyoxirane radical ring opening TS, C2 *anti* radical to *cis* vinoxy, OH rotated “out” from ring
H G4 -228.21393
H W1BD -228.38090
C,0,0.3884805216,-0.4164308439,0.3414897981
H,0,0.6348137986,-0.714940149,1.3603549344
C,0,-0.9163316666,-0.5399873059,-0.1936487188
H,0,-1.4471540881,-1.1872176238,-0.877190173
O,0,-1.1252906739,0.7120883592,0.1095214359
O,0,1.373361979,0.189789575,-0.3245133072
H,0,2.0315491294,0.5492139884,0.2880600305

TS-B Hydroxyoxirane radical ring opening TS, C2 *anti* radical to *trans* vinoxy, OH rotated “in” towards ring
H G4 –228.23251
H W1BD -228.39658
C,0,0.3464922868,-0.1385041124,0.4511476173

H,0,0.3607133361,-0.9282643647,1.1872785539
C,0,-0.8074604596,0.6332961065,0.2297777893
H,0,-0.9759885118,1.6452225126,-0.1226811804
O,0,-1.2471845451,-0.487932264,-0.3444837893
O,0,1.498483931,-0.0601693289,-0.2101474447
H,0,1.4715279626,0.6875884509,-0.8232785461

TS-B Hydroxyoxirane radical ring opening TS, C2 *anti* radical to *trans* vinoxy, +1.5 kcal/mol, OH rotated “out” from ring
H G4 -228.23030
H W1BD -228.39417
C,0,0.3420464245,-0.057578369,0.433970672
H,0,0.3776330515,-0.7719069582,1.2468698658
C,0,-0.8415230251,0.6427681214,0.1610198268
H,0,-1.043928304,1.5950340743,-0.3125292465
O,0,-1.2481075774,-0.5532530675,-0.2725363768
O,0,1.4519130519,0.0835760911,-0.2885620532
H,0,2.0890093786,-0.6132618921,-0.0806676882

TS-A Fluorooxirane radical ring opening TS, C2 *syn* radical to *trans* vinoxy
H G4 -252.24239
H W1BD -252.44942
C,0,0.33840943,-0.03111504,0.4227791832
H,0,0.4040155974,-0.6734134857,1.2897240956
C,0,-0.7949978906,0.6549076728,-0.0025980426
O,0,-1.2233288534,-0.5767101724,-0.1997451564
F,0,1.4884560139,-0.0052728915,-0.2512745856
H,0,-1.3256162972,1.5908479168,0.0528745057

TS-B Fluorooxirane radical ring opening TS, C2 *syn* radical to *cis* vinoxy
H G4 -252.22985
H W1BD -252.43872
C,0,-0.3640139012,0.4597977969,0.3207308933
H,0,-0.6982242536,0.8817831027,1.2649297869
C,0,0.8998694968,0.5287315904,-0.2424693022
H,0,1.6394757997,1.2306241118,-0.5915821013
O,0,1.0243078486,-0.7173868147,0.1707557047
F,0,-1.3601969902,-0.2165177871,-0.2571689815

TS-A Fluorooxirane radical ring opening TS, C2 *anti* radical to *cis* vinoxy
H G4 -252.23200
H W1BD -252.43893
C,0,-0.368760162,0.4525786423,0.3256489045
C,0,0.9041887311,0.5254522953,-0.2350675235
O,0,1.016726101,-0.7217850062,0.1478528302
H,0,1.5309927605,1.1292051672,-0.8720166325
H,0,-0.6887993863,0.8608896375,1.27870715
F,0,-1.3663200444,-0.240346736,-0.2406377287

TS-B Fluorooxirane radical ring opening TS, C2 *anti* radical to *trans* vinoxy
H G4 -252.24203

H W1BD -252.44954
C,0,0.3410872086,-0.0880507811,0.4226764409
H,0,0.4076026963,-0.8152164008,1.2182282354
C,0,-0.7887102698,0.6591551977,0.1338440333
H,0,-1.0475924567,1.6366783272,-0.2401191299
O,0,-1.2415642897,-0.5334303753,-0.2564163884
F,0,1.4780501114,-0.0059649676,-0.2660291914

TS-A 1,1-Difluorooxirane radical ring opening TS
H G4  failed; either NIMAG=2 or radical carbon inverted
H W1BD failed; either NIMAG=2 or radical carbon inverted

TS-B 1,1-Difluorooxirane radical ring opening TS
H G4 -351.46781
H W1BD -351.78344
C,0,-0.2563558968,-0.0546141509,0.1689310088
C,0,1.0333007168,-0.3378545217,0.5769857223
O,0,1.3943596617,0.1994015352,-0.5872822672
F,0,-1.1348318224,-0.9285374275,-0.2581743987
H,0,1.5053970891,-1.1405119944,1.1249704873
F,0,-0.7984327483,1.1328985594,0.1487674475

TS-A Trifluorooxirane radical ring opening TS
H G4 -450.68964
H W1BD -451.11382
C,0,0.5701963858,-0.0940011029,-0.1638077537
C,0,-0.8185934433,-0.310381612,0.1771370051
O,0,-0.6501036013,0.4595702882,1.1784667781
F,0,-1.8563107774,-0.2045353645,-0.66770203
F,0,1.4800165776,-0.974213375,0.1019720519
F,0,1.1196148586,1.0418401663,-0.4941330513

TS-B Trifluorooxirane radical ring opening TS
H G4 -450.69328
H W1BD -451.11670
C,0,-0.5528600955,0.0935491234,-0.1148501819
C,0,0.7793379474,-0.3303448999,-0.2945255899
O,0,0.619089493,-1.1176799935,0.7392757939
F,0,1.7915235023,0.5590711808,-0.3622433868
F,0,-0.9186478954,1.1721042136,0.4999615236
F,0,-1.5734759516,-0.5784326244,-0.522021159

TS-A Chlorooxirane radical ring opening TS, C2 *syn* radical to *trans* vinoxy
H G4 not obtained: optimization converged, but failed on CCSD step
H W1BD -614.02238
C,0,-0.1997827011,-0.0087706667,0.531018653
H,0,-0.2602865076,-0.5629082307,1.4549887202
C,0,-1.2752996954,0.6518950336,-0.0647947743
O,0,-1.6387468999,-0.5963665399,-0.2688509806
H,0,-1.8430265465,1.5680457289,-0.0667864155
Cl,0,1.4130153504,-0.0139803252,-0.1266952027

TS-B Chlorooxirane radical ring opening TS, C2 *syn* radical to *cis* vinoxy
H G4 failed; either NIMAG=2 or radical carbon inverted
H W1BD failed; either NIMAG=2 or radical carbon inverted

TS-A Chlorooxirane radical ring opening TS, C2 *anti* radical to *cis* vinoxy
H G4 -612.49466
H W1BD -614.01296
C,0,0.1966629957,0.6235506545,0.3398489634
C,0,1.4116437112,0.4091976252,-0.3311746696
O,0,1.3803349817,-0.7694954158,0.2176908283
H,0,1.9406080734,0.7437093395,-1.2129360923
H,0,0.0953836743,1.1980948902,1.2507411252
Cl,0,-1.3321904363,-0.1086080936,-0.109379155

TS-B Chlorooxirane radical ring opening TS, C2 *anti* radical to *trans* vinoxy
H G4 -612.50337
H W1BD -614.02273
C,0,-0.200958172,-0.0896685309,0.5463754523
H,0,-0.253777961,-0.7816542098,1.3710893849
C,0,-1.287758548,0.6568610439,0.1120593743
H,0,-1.4873333007,1.6297788576,-0.3087563536
O,0,-1.6564358079,-0.5374523971,-0.3481831519
Cl,0,1.4001387896,0.0005222363,-0.1360967061

**C2 cyclopropyl radical ring opening transition states**
Methycyclopropyl C2 radical ring opening TS, C2 *syn* radical to *cis* allyl radical, conformer 1
H G4 -156.35992
H W1BD -156.42668
C,0,0.3448807749,-0.4096034509,0.401773516
H,0,0.3589731909,-0.8111938756,1.4076319776
C,0,-0.8672773019,-0.607947669,-0.3918182466
H,0,-1.4710151831,-1.5042174277,-0.3786328365
C,0,-1.3263656721,0.6245965071,0.0382409379
H,0,-2.2938685257,0.7743467675,0.5177856556
H,0,-0.7609979792,1.529797304,-0.1432993128
C,0,1.5884007724,0.2168026437,-0.1333535429
H,0,2.2302020053,0.6095732666,0.6552747915
H,0,2.1673211383,-0.5224407087,-0.7030135475
H,0,1.3629637802,1.026671643,-0.8316703923

Methycyclopropyl C2 radical ring opening TS, C2 *syn* radical to *cis* allyl radical, conformer 2
H G4 -156.35584
H W1BD -156.42247
C,0,1.2284425555,0.73058745,0.0920455791
H,0,1.8311288192,0.8412533338,0.9839200895
H,0,0.8379294848,1.6301171621,-0.3575302116
C,0,0.9000589398,-0.6134392044,-0.3919418193
H,0,1.5795228729,-1.4404688549,-0.2322424266
C,0,-0.3287642893,-0.580813816,0.2499386377
H,0,-0.5543139842,-1.3407776434,1.0045262861

C,0,-1.5091989095,0.2828830387,-0.0894733592
H,0,-2.3235773883,-0.3726872158,-0.4175801556
H,0,-1.8819887943,0.8352583776,0.7748261048
H,0,-1.2945233068,0.9830273722,-0.8933557249

Methylcyclopropyl C2 radical ring opening TS, C2 *anti* radical to *trans* allyl radical, conformer 1
H G4 -156.35735
H W1BD -156.42421
C,0,-0.3645816446,-0.0692031931,0.554524858
H,0,-0.3940726064,0.2220254538,1.59913408
C,0,0.8260908195,-0.7503546825,0.0323656258
H,0,0.7967845281,-1.5268995474,-0.7192002561
C,0,1.443997562,0.4698162941,-0.1543438432
H,0,1.8609568824,0.8055197974,-1.1051757615
H,0,1.6004591824,1.1464030239,0.6770293751
C,0,-1.5912587548,0.1553994841,-0.2679494674
H,0,-2.366517179,-0.5824523434,-0.0207803711
H,0,-2.0331430389,1.1418546528,-0.09908805
H,0,-1.3809307508,0.0507790602,-1.3327291897

Methylcyclopropyl C2 radical ring opening TS, C2 *anti* radical to *trans* allyl radical, conformer 2
H G4 -156.36201
H W1BD -156.42836
C,0,-1.5806548689,-0.3305503825,0.2459132342
H,0,-1.6953667481,-0.5010441544,1.308155498
H,0,-2.0926000239,-1.0048628003,-0.4267467119
C,0,-0.717458265,0.7499334067,-0.2377640348
H,0,-0.5756934863,1.673292888,0.3088800128
C,0,0.3096924962,-0.1624674433,-0.4071874053
H,0,0.1584773429,-0.987692671,-1.0958869583
C,0,1.6775338692,-0.0881742623,0.2059098519
H,0,2.4427533736,-0.1390426687,-0.5738569795
H,0,1.814672879,0.8282275739,0.7780802729
H,0,1.8542334314,-0.9373854862,0.8704992201

Fluorocyclopropyl C2 radical ring opening TS, C2 *syn* radical to *cis* allyl radical, conformer 1
H G4 -216.30154
H W1BD -216.46051
C,0,-0.4231161924,0.3018830948,0.4013727475
H,0,-0.6124119629,0.5250336946,1.4407697887
C,0,0.7511899735,0.6484059185,-0.3453015815
H,0,1.26120219,1.5984835195,-0.2913101601
C,0,1.2998502683,-0.5739910616,0.0205551095
H,0,2.2725621761,-0.6799812897,0.5007240317
F,0,-1.4965042937,-0.2513602827,-0.2054502619
H,0,0.8030588411,-1.5025525934,-0.2326676739

Fluorocyclopropyl C2 radical ring opening TS, C2 *syn* radical to *cis* allyl radical, conformer 2
H G4 -216.29774
H W1BD -216.45671
C,0,-1.1930260664,-0.6678292607,0.0884346484

H,0,-1.8870250877,-0.7312958084,0.9154535311
C,0,-0.7673287418,0.6538192536,-0.393396466
H,0,-1.3614398892,1.5275934463,-0.163590298
C,0,0.4355935032,0.5132596075,0.251716089
H,0,0.8316947613,1.1436400216,1.0439469603
H,0,-0.7795355625,-1.5827257231,-0.3027995615
F,0,1.3797660831,-0.3705485367,-0.1320769033

Fluorocyclopropyl C2 radical ring opening TS, C2 *anti* radical to *cis* allyl radical, conformer 1
H G4 -216.29828
H W1BD -216.45752
C,0,0.4579268961,0.1664197326,0.5061870935
H,0,0.6715155851,-0.0986350873,1.5308681505
C,0,-0.7444441735,0.7619547683,-0.0160439361
H,0,-0.7622752339,1.4959139749,-0.8077242502
C,0,-1.3390078717,-0.4882315495,-0.0763577196
H,0,-1.7538679139,-0.9212664703,-0.9879280547
H,0,-1.4598677312,-1.0929653925,0.8140393707
F,0,1.4456984431,-0.2243969763,-0.3414986541

Fluorocyclopropyl C2 radical ring opening TS, C2 *anti* radical to *cis* allyl radical, conformer 2
H G4 -216.30859
H W1BD -216.46723
C,0,-1.4952729127,-0.3162950483,0.2224746498
H,0,-1.7009059429,-0.4054944136,1.2806959403
H,0,-1.9778329635,-1.0177581778,-0.44370387
C,0,-0.6137851832,0.7526958907,-0.2613367272
H,0,-0.4694356928,1.6903138782,0.2544295093
C,0,0.375957732,-0.1885150678,-0.3568187944
H,0,0.3599087075,-1.0562613118,-1.0011219934
F,0,1.5703802557,-0.0811957495,0.2626762856

1,1,2-Trifluorocyclopropane C2 radical ring opening, conformation 1
H G4 -414.74353
H W1BD -415.11974
C,0,0.581976453,-0.0175205754,-0.0343566488
C,0,-0.7944928806,0.08124235,-0.3143824594
C,0,-0.8569213812,1.3252842078,0.2973200185
H,0,-1.4554361881,1.5242423432,1.1871316018
H,0,-0.3993420764,2.1803454741,-0.1841927278
F,0,1.5570201788,0.0861352382,-0.9353923181
F,0,1.0802031707,-0.5248395421,1.0938821697
F,0,-1.7132672762,-0.8996604959,-0.226085636

1,1,2-Trifluorocyclopropane C2 radical ring opening, conformation 2
H G4 -414.74970
H W1BD -415.12475
C,0,0.7865278033,1.3357632045,0.5260894536
H,0,1.1930208136,1.1660058141,1.515346519
H,0,0.4184872083,2.313877758,0.2607371462
C,0,0.7542658788,0.226293851,-0.4156062643

C,0,-0.5473346739,-0.0862393597,-0.0600134209
F,0,1.6815609342,-0.7548769245,-0.3204980727
F,0,-1.6069275126,0.5774369167,-0.4688532631
F,0,-0.9183694516,-1.1864532601,0.5570039022
